# Virtual Reality Applications in Medicine During the COVID-19 Pandemic: Systematic Review

**DOI:** 10.2196/35000

**Published:** 2022-10-25

**Authors:** Federica Pallavicini, Alessandro Pepe, Massimo Clerici, Fabrizia Mantovani

**Affiliations:** 1 Department of Human Sciences for Education Università degli Studi di Milano-Bicocca Milano Italy; 2 Gamers VR Lab Università degli Studi di Milano-Bicocca Milano Italy; 3 Department of Medicine and Surgery Università degli Studi di Milano-Bicocca Monza Italy

**Keywords:** virtual reality, medicine, mental health, physical health, education, training, COVID-19

## Abstract

**Background:**

Virtual reality can play an important role during the COVID-19 pandemic in the health care sector. This technology has the potential to supplement the traditional in-hospital medical training and treatment, and may increase access to training and therapies in various health care settings.

**Objective:**

This systematic review aimed to describe the literature on health care–targeted virtual reality applications during the COVID-19 crisis.

**Methods:**

We conducted a systematic search of the literature on the PsycINFO, Web of Science, and MEDLINE databases, according to the Preferred Reporting Items for Systematic Reviews and Meta-Analysis guidelines. The search string was as follows: “[(virtual reality)] AND [(COVID-19) OR (coronavirus) OR (SARS-CoV-2) OR (healthcare)].” Papers published in English after December 2019 in peer-reviewed journals were selected and subjected to the inclusion and exclusion criteria. We used the Mixed Methods Appraisal Tool to assess the quality of studies and the risk of bias.

**Results:**

Thirty-nine studies met the inclusion criteria. Seventeen studies showed the usefulness of virtual reality during the COVID-19 crisis for reducing stress, anxiety, depression, and pain, and promoting physical activity. Twenty-two studies revealed that virtual reality was a helpful learning and training tool during the COVID-19 crisis in several areas, including emergency medicine, nursing, and pediatrics. This technology was also used as an educational tool for increasing public understanding of the COVID-19 pandemic. Different levels of immersion (ie, immersive and desktop virtual reality), types of head-mounted displays (ie, PC-based, mobile, and standalone), and content (ie, 360° videos and photos, virtual environments, virtual reality video games, and embodied virtual agents) have been successfully used. Virtual reality was helpful in both face-to-face and remote trials.

**Conclusions:**

Virtual reality has been applied frequently in medicine during the COVID-19 pandemic, with positive effects for treating several health conditions and for medical education and training. Some barriers need to be overcome for the broader adoption of virtual reality in the health care panorama.

**Trial Registration:**

International Platform of Registered Systematic Review and Meta-analysis Protocols (INPLASY) INPLASY202190108; https://inplasy.com/inplasy-2021-9-0108/

## Introduction

### Background

On March 11, 2020, the World Health Organization declared the spread of the novel coronavirus (COVID-19) a global pandemic [1]. As a primary measure to control the spread of the virus, many governments worldwide recommended staying at home and practicing social distancing, dramatically affecting people’s daily lives [2-4]. Health care represents one of the sectors most affected by the adverse effects of the COVID-19 pandemic [5]. Prolonged rules of social distancing and stay-at-home directions caused relevant difficulties in carrying out several in-hospital clinical activities [6,7], and the sudden interruption of medical education and training programs [8-10].

In such times of isolation and limited resources due to the COVID-19 crisis, information and communication technologies (ICTs) have empowered medical institutions to meet mental health and learning needs with scalable solutions [11,12]. Virtual reality (VR) represents an ICT development with the potential to revolutionize clinical support and education [13-17].

By definition, VR is a set of technologies, including head-mounted displays (HMDs), computers, and mobile devices, that can immerse users in a 3D environment to different degrees [18-21], from a simple presentation on a 2D display screen system (ie, desktop VR) up to highly immersive systems (ie, immersive VR) that use HMDs.

VR in the last decade has become a game changer for the health care sector in more than one way [22-25], representing a helpful instrument both for the treatment of several health conditions and for medical education and training [22,25-28]. This technology has been successfully applied to a wide range of mental disorders [29,30], including anxiety [31-33] and depression [34]. Furthermore, VR is being used in physical rehabilitation for improving motor function, fitness, movement quality, and mobility [35,36], and it has also been adopted as an enjoyable method for managing pain [37-40]. Regarding education and training, VR was greatly appreciated by medical and nursing students [41-43], and proved to play a crucial role in improving medical knowledge [44-46] and fostering surgical skills [44,46,47].

Therefore, during the COVID-19 crisis, VR has the potential to supplement the traditional in-hospital medical training and treatment [48], and may increase access to training and therapies in various health care settings [49,50].

### Research Question

Since, to the best of our knowledge, no previous work has investigated the use of health care–targeted applications of VR during the COVID-19 crisis, this systematic review aimed to describe the literature on this topic.

## Methods

### Databases Searched

A systematic search of the literature was performed on March 3, 2022, by 2 of the authors (FP and AP) following the Preferred Reporting Items for Systematic Reviews and Meta-Analysis (PRISMA) guidelines [51]. The review was preregistered (September 29, 2021) in the International Platform of Registered Systematic Review and Meta-analysis Protocols (INPLASY; INPLASY202190108). The search databases were PsycINFO, Web of Science, and MEDLINE.

### Inclusion Criteria

Two authors (FP and AP) established clear inclusion criteria to determine papers’ eligibility for inclusion in the review. Only studies meeting the following criteria were considered eligible for inclusion: (1) type of participant: all human participants (clinical and nonclinical population); (2) intervention: VR; (3) comparators: usual intervention, non-VR group, or none; (4) outcomes: focused on health care–targeted applications of VR during the COVID-19 pandemic; and (5) study design: randomized controlled trial (RCT), quantitative nonrandomized study (eg, nonrandomized controlled trial and case-control study), quantitative descriptive study (eg, survey, case series, and case report), or mixed methods study.

Papers published in English between December 2019 and March 2022 in peer-reviewed journals were selected and subjected to the inclusion criteria outlined above.

### Exclusion Criteria

Studies were excluded if they (1) did not use VR; (2) did not focus on the applications of VR for the treatment of health conditions, or for medical education and training during the COVID-19 pandemic; (3) did not describe research conducted after the outbreak of the COVID-19 crisis; (4) did not specify the period when the study was conducted; (5) did not include outcome measures or did not include complete results; (6) did use only qualitative data; and (7) were letter to the editor articles, commentaries, or reviews.

### Search Terms and Selection of Papers for Inclusion

The search string was as follows: “[(virtual reality)] AND [(COVID-19) OR (coronavirus) OR (SARS-CoV-2) OR (healthcare)].”

Initially, 2 authors (FP and AP) checked the titles and abstracts of identified articles to determine their eligibility. Subsequently, they independently reviewed the full text of potentially eligible papers. A consensus between the authors (FP and AP) resolved disagreements. When papers provided insufficient data for inclusion in the analysis, the corresponding authors were contacted to provide additional data. Five additional articles emerged via hand-searching and reviewing the reference lists of relevant papers.

### Study Quality and Risk of Bias Assessment

We used the Mixed Methods Appraisal Tool (MMAT) [52] to assess the methodological quality of the studies included and the risk of bias. Studies could be awarded a score of 0, 25, 50, 75, or 100 (with 100 being the highest quality). The MMAT has high reliability and efficiency as a quality assessment protocol and can concomitantly appraise methodological quality across various empirical research [53]. Two authors (FP and AP) independently assessed the studies’ quality. Interrater reliability was calculated with Cohen kappa [54], using the software package SPSS (IBM Corp).

### Data Extraction

Two authors (FP and AP) independently extracted the following data.

#### Study Characteristics

The study characteristics included (1) the *study outcomes* (treatment, education, and training); (2) the *study design* used (RCT, quantitative nonrandomized study, quantitative descriptive study, or mixed methods study); (3) the *populations* included in the study (sample size, profession or health condition, gender, mean age or age range, and nationality); and (4) the *measures used for the assessment of outcomes* (eg, self-report questionnaires, semistructured interviews, and users’ session data). An indication of the mean age or age range identified studies conducted on children (ie, under 12 years old), adolescents (12-18 years old), young adults (18-35 years old), middle-aged adults (36-55 years old), and older adults (over 55 years old). The division in these age ranges followed previous studies [55-57]. Regarding the study outcomes, we divided the selected papers into 2 main domain-specific categories related to VR applications in health care: (1) treatment, and (2) education and training.

#### VR Characteristics

The VR characteristics included (1) the *level of immersion* (high, medium, or low); (2) in the case of immersive VR, the specific *type of HMD* (ie, PC-based, console-based, mobile, or standalone system); (3) the *content* (virtual environments, 360° photos and videos, embodied virtual agents, VR video games, social VR platforms, or hybrid); (4) the *site of use* (face-to-face or remotely), the *user mode* (single user or multiuser); and (5) the *time of use* (the total amount of sessions and VR duration of use). The level of immersion, defined as a quantifiable feature of a technology that includes the extent to which it is possible to immerse oneself in the virtual world through interfaces [58], was considered because, based on it, it is possible to distinguish VR in different categories [18-21] (Table 1). Second, in the case of studies where an HMD was used, the model was specified to describe its specific type based on its implemented technologies (Table 1). Third, since there are different types of experiences in VR, information on the content was included (Table 1). Fourth, the site of use was considered since it represents crucial information concerning how VR can be offered. Fifth, information on user mode was included since VR content can be single user (ie, usable by a single user) or multiuser (ie, 2 or more users can share the same VR experience and communicate or interact with it). Finally, in the studies that indicated the time of VR use, this information was included, offering valuable insights about how and how long to use this technology to treat mental and physical health conditions, and in medical education and training.

**Table 1:** Classification of the level of immersion in virtual reality (VR) systems [18-21], the types of head-mounted displays, and the VR content.

Variable			Definition		Hardware/examples
**Immersion**					
	High (immersive VR^a^)	The user is immersed within a 3D content.		HMDs^b^	
	Medium (semi-immersive VR)	Rooms within which computer-generated environments are projected onto the walls.		C-Automatic Virtual Environment (CAVE)	
	Low (desktop VR)	Computer-generated environments made in 3D but which are shown on 2D displays.		PC, television, mobile phone, or tablet screen	
**Types of HMDs**					
	PC-based	Requires a connection between an HMD and a computer with advanced computational and graphics capabilities.		Oculus Rift S and HTC Vive	
	Console-based	Needs a connection between an HMD and a specific game console.		PlayStation VR	
	Mobile	Involves the integration of VR systems on mobile devices thanks to specific HMDs.		Samsung Gear VR and low-cost HMDs compatible with mobile phones, such as Google Cardboard	
	Standalone (all-in-one)	They do not need other technologies to work.		Meta Quest II, HTC Vive Focus, and Pico Interactive Neo	
**Content**					
	Virtual environments	Simulation on a computer of 3D environments, which can be explored in real-time and in which the user can interact with objects contained within it [59]. They are created through specific software such as Unreal or Unity.		N/A^c^	
	360° videos and photos	Images or videos of real or digital environments presented in a spherical version. They provide a spherical view with multiple viewing angles and perspectives. The contents are omnidirectional and can either be computer generated or captured from the real world [60].		YouTube VR and Google Earth VR	
	Embodied virtual agents	Graphical representations of the individuals controlled by the computer itself using an artificial intelligence program [61].		N/A	
	VR video games	Video games played through HMDs in which the player can interact with virtual content not only through a joypad or a keyboard, but also using head rotation, eye movements, or specially designed controllers that respond to the position and movements of the player in a defined space [62]. They include the following: - Commercial off-the-shelf video games that are “games that one can purchase on the high street” [63], or rather purchasable in online or physical stores - Custom-made games, often defined in the literature as “serious games” [64], that are games created ad hoc by researchers to educate, train, or change behavior		Beat Saber, Half-Life: Alyx, Superhot VR, and Fruit Ninja VR	
	Social VR platforms	3D virtual spaces where multiple geographically remote users can interact with one another through VR HMDs [65,66]. At present, several online VR applications with a social component exist.		AltspaceVR, Horizon Worlds, VRChat, and VRzone	

^a^VR: virtual reality.

^b^HMD: head-mounted display.

^c^N/A: not applicable.

## Results

### Overview

**Figure 1:**
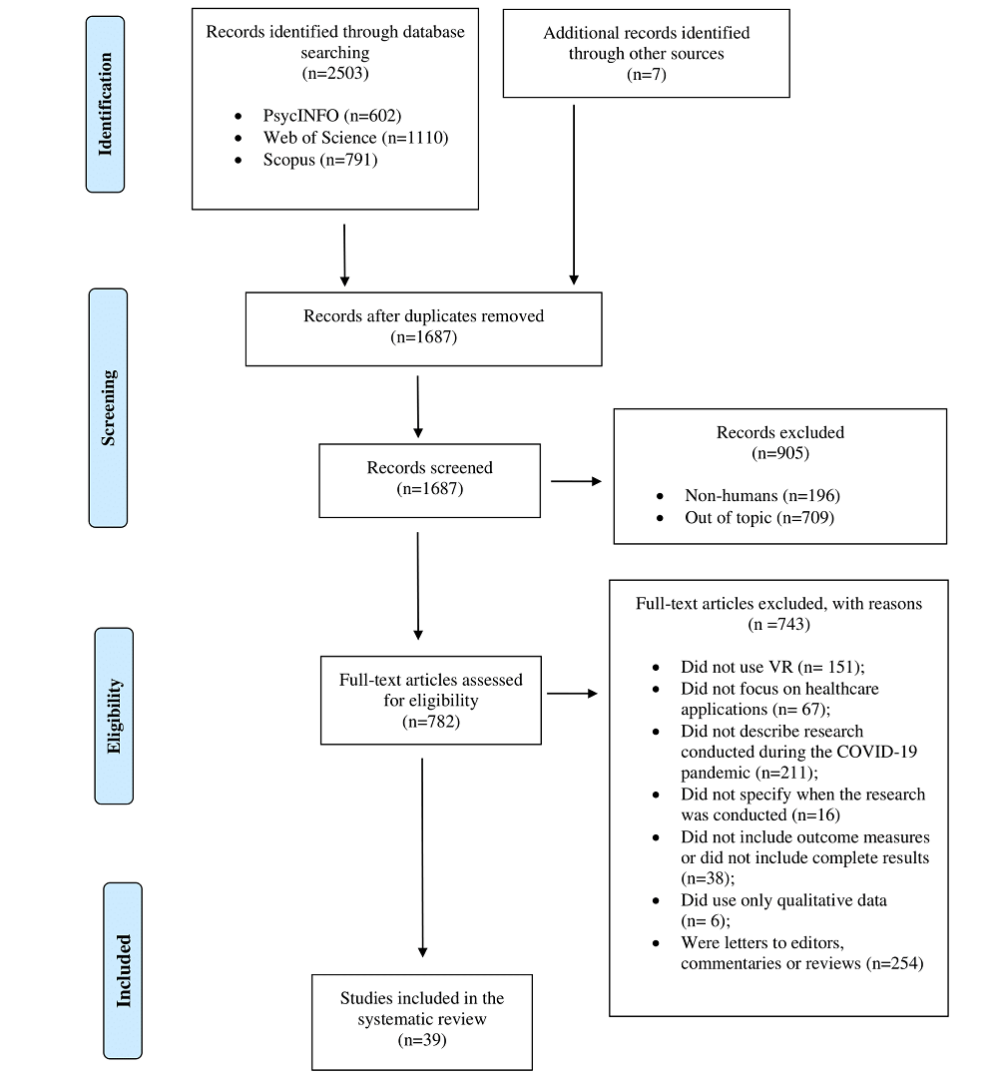
Preferred Reporting Items for Systematic Reviews and Meta-Analysis (PRISMA) flow chart. VR: virtual reality.

The search strategy retrieved 2503 records published after December 2019. A total of 1687 studies remained after deduplication and language examination, and 905 records were excluded after the first screening, and title and abstract analysis. Full-text copies of the 782 remaining studies were obtained and subjected to further evaluation. After reading the full-text copies, 743 studies were excluded based on our exclusion criteria, resulting in 39 studies being included in our systematic review (Figure 1).

### Quality Assessment Outcomes

Interrater reliability was 0.827, representing substantial agreement [67]. Within this systematic review, the distribution of MMAT scores varied significantly with the study design (Table 2). Nineteen studies (49%) met the MMAT quality assessment score of 75% or above, implying that much of the research in this area is of high quality (Multimedia Appendix 1). Nevertheless, the quality scores varied substantially according to the study design.

**Table 2:** Study design and Mixed Methods Appraisal Tool score distribution.

MMAT^a^ score distribution			References		Value, n (%)
**Quantitative randomized controlled trial (N=4)**					
	0	N/A^b^		0 (0)	
	25	N/A		0 (0)	
	50	[68,69]		2 (50)	
	75	N/A		0 (0)	
	100	[70,71]		2 (50)	
**Quantitative nonrandomized study (N=10)**					
	0	N/A		0 (0)	
	25	[72]		1 (10)	
	50	[73-77]		5 (50)	
	75	[78-81]		4 (40)	
	100	N/A		0 (0)	
**Quantitative descriptive study (N=19)**					
	0	N/A		0 (0)	
	25	[48,82-84]		4 (21)	
	50	[49,85-89]		6 (32)	
	75	[90-94]		5 (26)	
	100	[95-98]		4 (21)	
**Mixed methods study (N=6)**					
	0	[99]		1 (17)	
	25	N/A		0 (0)	
	50	[100]		1 (17)	
	75	[101,102]		2 (33)	
	100	[103,104]		2 (33)	

^a^MMAT: Mixed Methods Appraisal Tool.

^b^N/A: not applicable.

### Study Characteristics

#### Study Outcomes

Seventeen studies focused on the applications of VR during the COVID-19 pandemic to treat different health conditions, while 22 studies investigated its use for medical education and training (Multimedia Appendix 2).

#### Treatment

Ten studies showed the usefulness of VR for reducing stress, anxiety, and depression during the COVID-19 pandemic, and there were several findings. First, a self-administered at-home VR-based intervention through low-cost HMDs was useful during the COVID-19 lockdown for diminishing depression levels, stress levels, general distress, and perceived stress in healthy individuals up to the 2-week follow-up [74]. Second, immersive VR experiences showing calming nature scenes (ie, VRelax and Tranquil Cinematic-VR simulation) were helpful in reducing perceived stress among COVID-19 intensive care unit (ICU) health care workers [80,81]. Third, guided meditation and exploration of natural environments using immersive VR were highly satisfactory for patients recovering in a COVID-19 ICU, with perceived benefits in coping with isolation and loneliness [49]. The ICU staff considered VR logistically and operationally feasible [49]. Fourth, remote reminiscence training conducted during the first months of the COVID-19 pandemic using immersive VR diminished state anxiety without serious side effects (ie, nausea, dizziness, and headache) in patients with mild cognitive impairment [91]. Fifth, immersive VR exposure therapy showing scenes related to COVID-19 was effective in diminishing anxiety symptoms among patients with chief complaints of fear of COVID-19 [85], and in reducing levels of posttraumatic stress disorder (PTSD), anxiety, and depression among patients treated in the ICU due to COVID-19 [96]. The use of VR in the ICU was considered by patients to be feasible, and improved their satisfaction with and ratings of the ICU after care [71]. Sixth, immersive VR showing 360° YouTube videos reduced depressive symptoms in an adult with mild depressive disorder [95]. Seventh, 360° virtual tourism experiences diminished perceived stress among healthy young adults [92].

VR has also been used as a distraction tool for pain management during the COVID-19 pandemic. A self-administered daily at-home immersive VR-based program (ie, EaseVRx) helped reduce pain intensity and interference with activity, stress, mood, and sleep over time among young adults experiencing chronic low back pain [70].

Four studies reported the efficacy of VR for promoting physical activities during the COVID-19 crisis. First, a desktop VR video game developed ad hoc and used at home improved movement performance and physical activity intensity among patients with cerebral palsy [79]. Second, commercial off-the-shelf VR video games (ie, “Box VR” and “NVIDIA VR Fun House”) fostered physical exercise in 4 older people without adverse effects [90]. Participants reported that using such games was “positive and relaxing, and motivates you to exercise” [90]. Third, VR recreational use significantly increased during the COVID-19 lockdown period, and users expressed overwhelmingly positive opinions on the impact of VR activities on their mental and physical health. The self-reported intensity of physical activity was considerably more strenuous in VR users than in console users [86]. Fourth, VR fitness impacted individuals’ physical and mental health, playing a substantial role in improving the overall quality of life and positively influencing the behavior and attitude of users [94].

Two studies provided early indications that Spanish-speaking (ie, Spain and Latin America) therapists have begun using VR to help their patients during social isolation due to COVID-19 [97,98], and 20% of the therapists indicated that they had used VR technology for remote psychological support [98].

#### Education and Training

Six studies revealed that VR represented a helpful learning and training tool during the COVID-19 pandemic in several areas. First, watching 360° videos on desktop displays effectively taught emergency medicine during the COVID-19 crisis [102]. Second, immersive VR showed relatively better outcomes regarding skills acquired, learning speed, and information retention rates than classroom training in a sample of frontline health workers [100]. Third, pediatric residents experienced a desktop VR-based training program as immersive, feasible, and realistic in terms of the clinic setting, and as a safe space to practice and learn new skills [101]. Fourth, VR-based pregraduation medical training was considered realistic with regard to the initial clinical assessment and diagnostic activity by medical students [48], even if a nonnegligible proportion of the students experienced difficulties in online access to the VR platform [48]. Fifth, an immersive social VR platform was reported as easy to use, helpful, and better than tele and video conferencing for remote multidisciplinary heart team meetings [82]. Sixth, applicants of a radiology residency reported positive attitudes toward a nonimmersive social VR platform [83].

Six studies successfully adopted VR for teaching various medical topics during the COVID-19 pandemic, and there were several findings. First, immersive VR was useful for teaching brain and spinal cord neuroanatomy and for practicing neurorehabilitation exercises [93]. Second, desktop VR–based training was used successfully for teaching nursing content [72,75,78] and urology [84]. Third, immersive VR experiences helped simulate pediatric ICU clinical scenarios, with some specific critiques regarding limited realism in some mechanical aspects of the simulation [99].

Six studies evaluated this technology to teach COVID-19–related skills to doctors and nurses, and as an educational tool for increasing public understanding of the COVID-19 crisis, and there were several findings. First, an immersive VR simulation (ie, COVID-19 VR Strikes Back) was at least as effective as traditional learning methods for training medical students regarding COVID-19–related skills [68]. Second, 2 immersive VR experiences, one involving wearing and stripping personal protective equipment [73] and the other involving the management of patients with respiratory infectious diseases due to COVID-19 [77], provided an effective and safe alternative to training nurses during the first year of the COVID-19 pandemic. Third, a virtual simulation of 2 patients (ie, COVID-19 and surgical trauma), which tried using desktop displays, helped nursing students bridge gaps in teaching and learning processes [104]. Fourth, a VR intervention using HMDs effectively increased COVID-19 vaccination intentions among unvaccinated young adults [69]. Fifth, VR, using desktop displays and HMDs, provided an effective educational tool for COVID-19 pandemic fundamental knowledge, increasing public understanding of the spread of the crisis [76].

Four studies investigated the adoption rate and the perception of VR in medicine during the COVID-19 pandemic. First, pediatric health care providers reported frequent modifications to existing simulation programs during the first months of the COVID-19 pandemic, including VR training [88]. Second, medical students mostly agreed that VR and online teaching compensated for the suspension of face-to-face medical education and reported that these technologies are the best alternatives to physical learning [89]. Third, the potential of VR for future teaching was rated low in a sample of medical students and lecturers, probably due to a lack of practical experience [87]. Fourth, high-fidelity immersive VR and specialized profession-specific resources were used heavily in medical education and training during the first year of the COVID-19 pandemic [103].

#### Study Design

Considering the entirety of the studies, the quantitative descriptive was the design of 19 studies (ie, 12 surveys and 7 case report studies). Ten studies adopted a quantitative nonrandomized design, 6 adopted a mixed methods design, and 4 adopted an RCT design (Table 2).

#### Populations

The number of participants ranged from 1 [95,96] to 4300 [94]. The study samples’ characteristics are described in Table 3.

**Table 3:** Study characteristics.

Characteristic				References		Value (N=39), n (%)
**Study outcome**						
	Treatment		[49,70,71,74,79-81,85,86,90-92,94-98]		17 (44)	
	Education and training		[48,68,69,72,73,75-78,82-84,87-89,93,99-104]		22 (56)	
**Sample**						
	**Students**					
		Medical students	[48,68,83,84,87,89,101,102]		8 (21)	
		Nursing students	[72,77,78,104]		4 (10)	
	**Health professionals**					
		Doctors	[82,88,93,99,103]		5 (13)	
		Nurses	[73,75]		2 (5)	
		COVID-19 frontline workers	[80,81,100]		3 (8)	
		Mental health professionals	[97,98]		2 (5)	
	**Patient health condition**					
		Recovered in the ICU^a^ due to COVID-19	[49,71,96]		3 (8)	
		Depression	[95]		1 (2)	
		Chronic back pain	[70]		1 (2)	
		Cerebral palsy	[79]		1 (2)	
		Mild cognitive impairment	[91]		1 (2)	
		Chief complaints of fear of COVID-19	[85]		1 (2)	
	**General population**					
		Healthy individuals	[69,74,76,86,90,92,94]		7 (18)	
**Age range**						
	Under 12 years old		N/A^b^		0 (0)	
	12-18 years old		[79]		1 (2)	
	18-35 years old		[68,69,72,74,75,78,80,86,87,89,92,94,101,104]		14 (36)	
	36-55 years old		[70,81,85,95,97,98]		6 (15)	
	Over 55 years old		[71,90,91,96]		4 (10)	
	Unspecified		[48,49,73,76,77,82-84,88,93,99,100,102,103]		14 (36)	
**Gender**						
	Both male and female		[68-72,74,75,77-82,85,86,89,91,92,94,97,98,101,102,104,105]		24 (62)	
	Male only		[90,95,96]		3 (8)	
	Female only		N/A		0 (0)	
	Unspecified		[48,49,73,76,83,84,87,88,93,99,100,103]		12 (31)	
**Nationality**						
	Europe		[48,68,69,71,74,81,82,84,86,87,89,90,96-98,102-104]		18 (46)	
	North America		[49,70,72,73,80,83,95,99,101]		9 (23)	
	South America		[79]		1 (2)	
	Asia		[75-78,85,91,92,94]		8 (20)	
	Africa		[93,100]		2 (5)	
	Oceania		N/A		0 (0)	
	Various		[88]		1 (2)	
**Outcome measures**						
	Self-report questionnaire		All		39 (100)	
	Performance task		[68,78,79]		3 (8)	
	Semistructured interview		[95,101,104]		3 (8)	
	User session data		[95,104]		2 (5)	
	Focus group		[104]		1 (2)	
	Knowledge task		[72,73,75-77,100]		6 (15)	
	Physiological data		[73]		1 (2)	

^a^ICU: intensive care unit.

^b^N/A: not applicable.

#### Outcome Measures

All the studies used self-reported questionnaires. Twenty-three studies adopted only this type of measure, while 16 studies also used other instruments, including performance tasks, semistructured interviews, knowledge tasks, users’ session data, focus groups, and physiological data (see Table 3 for details).

### VR Characteristics

Of the 39 studies included, 31 tested the efficacy of specific VR systems (Table 4), while 8 investigated the general use of VR in different samples (eg, mental health professionals and doctors) (Multimedia Appendix 3) [86-89,94,97,98,103].

**Table 4:** Virtual reality characteristics.

Characteristic					References			Value (N=31), n (%)
**Immersion**								
	High			[49,68-71,73,77,80-82,84,85,90,91,93,95,96,99,100]			19 (61)	
	Medium			N/A^a^			0 (0)	
	Low			[48,75,78,79,83,92,101,102,104]			9 (29)	
	Both high and low			[72,74,76]			3 (10)	
**Type of HMD^b^**								
	PC-based			[68,70,73,76,82,85,90,93]			8 (36)	
	**Console-based**							
		Mobile	[72,74,84]			3 (14)		
		Standalone	[49,69,71,77,80,81,91,95,99]			9 (41)		
	Unspecified			[96,100]			2 (9)	
**Content**								
	Virtual environments			[68,69,73,75-77,82,83]			8 (26)	
	360° videos or photos			[71,72,74,80,81,84,91,92,95,96,102]			11 (35)	
	Embodied virtual agents			[48,78,99,101,104]			5 (16)	
	VR^c^ video games			[79,90]			2 (6)	
	Hybrid			[49,70,93,100]			4 (13)	
	Unspecified			[85]			1 (3)	
**User mode**								
	Single user			[48,49,68-81,84,85,90-93,95,96,99-102,104]			29 (93)	
	Multiuser			[82,83]			2 (7)	
**Site of use**								
	Face-to-face			[49,68,70,71,73,76-78,80-82,84,85,90,92,93,96,99,100]			19 (61)	
	Remotely			[48,69,74,79,83,91,95,101,102,104]			10 (32)	
	Both			[72]			1 (3)	
	Unspecified			[75]			1 (3)	
**Time of use (in total)**								
	<10 minutes			[80,81,91-93,99]			6 (19)	
	11-60 minutes			[71,73,76-79,90,95,101]			9 (29)	
	61-180 minutes			[72,74,83,100]			4 (13)	
	>180 minutes			[48,70]			2 (6)	
	Unspecified			[49,68,69,75,82,84,85,96,102,104]			10 (32)	

^a^N/A: not applicable.

^b^HMD: head-mounted display.

^c^VR: virtual reality.

#### Level of Immersion

Nineteen studies used a VR system with high immersion (ie, immersive VR), while 9 tested a system with low immersion (ie, desktop VR). Three studies adopted both immersive and desktop VR systems [72,74,76]. No study used semi-immersive VR systems (Table 4).

#### Types of HMDs

Of the 22 studies that used an HMD, standalone systems were the most popular, with 9 studies using them. Eight studies adopted PC-based VR systems, 3 studies used mobile VR systems [72,74,84], and 2 studies did not specify the model of HMD [96,100] (Table 4).

#### Content

The most adopted VR content was 360° videos or photos, used in 11 studies. Eight studies used virtual environments, 2 studies adopted VR video games [79,90], 5 studies used embodied agents [48,78,99,101,104], 4 studies used hybrid content (ie, a mix of 360° videos or photo and virtual environments or VR video games) [49,70,93,100], and 1 study did not specify the content [85] (Table 4).

#### User Mode

The virtual content was of the single-user type in 29 studies and was of the multiuser type in 2 studies [82,83] (Table 4).

#### Site of Use

Nineteen studies used VR through face-to-face experiments, 10 used it remotely, and 1 used it both face-to-face and remotely [72]. Finally, 1 study did not specify the site of use [75] (Table 4).

#### Time of Use

In 21 studies reporting the number of sessions using VR, the mean number of sessions was 4.1, ranging from 1 (eg, [91,92,99]) to 56 sessions [70]. The number of minutes spent using VR differed among studies from about 5 minutes (eg, [79,99]) to up to 4 hours [48,70]. Ten studies did not indicate the exact time of use of VR (Table 4).

## Discussion

### Principal Findings

This systematic review examined studies conducted on VR applications in medicine during the COVID-19 pandemic, with a focus on using VR to treat health conditions and in medical education and training. After applying the inclusion criteria, 39 papers were included and analyzed.

From this systematic review, VR was found to be useful during the COVID-19 pandemic for reducing stress (eg, [75,80,96]), anxiety [91], depression [95], and pain [70], and promoting physical activity [79,90]. This technology has been successfully used in healthy people (eg, [74,86,92,94]); COVID-19 ICU health care workers [80,81]; patients with various diseases, including mild cognitive impairment [91], mild depressive disorders [95], and cerebral palsy [79]; chief complaints of fear of COVID-19 [85]; and individuals treated in the ICU due to COVID-19 [49,71,96].

VR has been successfully applied for decades to diminish stress, anxiety, depression, and pain [38,40,106-110]. Besides, this technology, especially immersive video games [111,112], stimulates physical activity [108], with long-term positive outcomes on mental and physical health [113,114]. What is new from the past, as noted in this review, is that VR is no longer used only in the specialist’s office or at the hospital but is also used remotely. The release of current standalone and low-cost mobile VR systems, thanks to the high ease of use and limited costs, has made this technology feasible for everyday in-home use [115]. Studies from this review showed that VR during the COVID-19 pandemic has begun to be adopted into mainstream clinical practice for remote psychological support [97,98]. This fact appears relevant since VR home-based training represents new promising interventions for remote support [98,116,117].

From this review, not only VR environments developed ad hoc, but also commercial contents (eg, VR video games downloaded from the Steam platform, and 360° videos and photos available on YouTube) were useful for diminishing stress [92], anxiety [91], and depression [95], and for promoting physical activity [90]. These findings seem interesting since using commercial off-the-shelf VR video games and experiences for promoting mental health and physical activity could have several advantages, including their low cost and ready-to-use format, their advanced graphic quality, and the possibility to reach millions of individuals worldwide [107,118].

The second main result of this systematic review is that during the COVID-19 pandemic, VR was a helpful learning tool for medical education and training in several areas, including emergency medicine, nursing, pediatrics, radiology, and cardiology (eg, [100-102]). This technology has been successfully used for teaching various topics, including neuroanatomy and clinical anatomy (eg, [84,93]). VR has also been adopted to train COVID-19–related skills among doctors and nurses [68,73]. Studies that emerged from this systematic review showed that, where available, high-fidelity immersive VR was adopted heavily after the outbreak of the COVID-19 pandemic [88,103].

Several previous studies proved the usefulness of VR for the training of medical students and nurses [43] and for teaching many medical topics and procedures, such as clinical anatomy [41,45] and radiation oncology [42]. This technology is considered a superior educational modality in comparison to passive teaching methods, such as learning from a textbook, slideshow, or lecture, thanks especially to the greater involvement of individuals in the learning process [119-121].

This review, with regard to treatment, showed that during the COVID-19 pandemic, VR started to be used for medical education and training in face-to-face sessions and remotely. This flexibility of access allowed the adoption of this technology into medical education curricula for both in-person and home-based activities [122]. Medical students mostly agreed that online teaching using VR compensated for the suspension of face-to-face medical education and reported this technology as the best alternative to physical learning [89].

Some studies that emerged in this review used content in multiuser VR, including social VR platforms [82]. Such VR environments are an emerging diverse group of multiuser online applications creating new opportunities for remote simulation and communication, and facilitating and extending the existing communication channels of the physical world [123,124]. Although its exploration has been limited so far, education using social VR will be increasingly relevant in the next few years. Indeed, major technology companies are making huge investments in the so-called “metaverse” (ie, a simulated digital environment that incorporates VR and other technologies, including augmented reality and artificial intelligence, to build spaces where users can interact as in the actual world) [125]. “Metaversities” could become the universities of the future [126].

In this systematic review, VR was also an effective educational tool for increasing public understanding and knowledge of the COVID-19 pandemic [76] and COVID-19 vaccination intentions [69]. This result, in line with previous literature [127,128], stresses the potential of VR as a useful and innovative tool for promoting scientific and medical knowledge among the general population. VR can improve health awareness and assist in health-related decision-making for the general population [129], increasing the overall motivation and engagement and leading to a more effective transfer of medical knowledge and comprehension of information [130,131]. In turn, this could improve individuals’ overall health, decrease hospitalization rates, and save long-term physician consultation costs [129].

Moreover, studies from this systematic review showed the usefulness of VR for treating health problems and for medical education and training in both the high (ie, immersive VR) and low immersion (ie, desktop VR) formats. This result appears important as these VR systems have very different characteristics and costs [132-134]. On the one hand, as emerged from studies included in this review (eg, [68,70]), immersive VR offers advantages over desktop VR [62,135]. However, desktop VR has the main advantages of being more readily usable and accessible, and having a lower cost.

Various types of HMDs (ie, PC-based, mobile, and standalone) and contents (ie, 360° videos and photos, virtual environments, VR games, and embodied virtual agents) have been used with positive results in medicine during the COVID-19 crisis (eg, [48,73,101]). This underscores how nowadays it is possible to choose among different VR hardware and software. To select the potentially most effective ones, it is critical to reason about the specific goal of VR use [136]. For example, in the case of VR for relaxation, it may be sufficient to include a low-cost mobile system and 360° videos or commercial off-the-shelf VR games. The same is true when VR is adopted to offer doctors and nurses “soft skills” (eg, problem-solving, communication, and interpersonal skills) training. On the other hand, in the case where VR is used for complex operations, simulation training, a PC-based system, haptic devices, and specially created virtual content should be used. In fact, in this case, the graphical and sensory fidelity of VR are crucial [137,138].

### Barriers for Using VR in the Health Care Sector

In addition to offering data in favor of the usefulness of VR in the health care sector, this review also raises some critical reflections on the possible limits of using this technology for these aims.

First, some health care workers experienced difficulties in using VR [48]. Due to a lack of knowledge of this technology, many individuals may find it challenging to use VR. To overcome the mentioned obstacle, governments and other societal bodies (eg, medical societies, medical schools, and residency training programs) should provide information about VR (eg, through training courses dedicated to health care practitioners and mental health professionals). They should offer clear guidelines for correctly using this technology in mental health, and medical education and training [15].

Second, when the graphic quality of the virtual experience was perceived as too low, users had difficulties comprehending a target scenario [73,87]. To prevent graphical or technical problems (eg, breakdown or problems with interaction) [139,140], with potential detrimental effects on treatment and learning outcomes [137,141], the principles and practices of user-centered design are recommended [142,143]. Many VR-based treatment, and medical education and training programs are currently taking a technology approach rather than a human-centered approach, which can lead to limited impact of VR in these fields. Due to the close bond between the user and the system within virtual environments, it may be impossible to segregate human factors from design issues when striving to achieve the potential of VR [144]. For this reason, it appears critical that a psychologist with expertise in human-computer interaction always be included in the team developing virtual experiences for the medical field.

Third, a primary issue in using this technology in the mental health panorama is related to its costs. The price of HMDs range from US $300 to US $1500. In the case of VR content developed ad hoc, the costs for implementation are high. One way to overcome the economic barrier of VR could be to use standalone or low-cost mobile systems [115,145] and, whenever possible, commercially available content [107,118]. Another solution could be providing hospitals with a certain number of VR systems to be available for their patients and staff for free [15].

### Recommendations for Future Research

More studies should be undertaken to expand the limited literature on home-based VR for treatment, and medical education and training. Multiuser VR platforms could be studied more deeply in education and as tools to implement future interventions for treating mental and physical health conditions. More research should be conducted to test the efficacy of commercial off-the-shelf VR experiences for treatment and in educational settings. Limitations and possibilities of VR systems with high versus low immersion require further investigation, for example, through RCT studies comparing their effectiveness for clinical or learning outcomes. It might be worth investigating the longer-term effects of VR on such outcomes. Finally, the number of desired or needed VR sessions and the specific time to spend using VR remain matters of debate.

### Limitations

This review summarizes health care-targeted applications of VR during the COVID-19 pandemic based on specific keywords used in the search string, the databases included, and the review’s time period. Therefore, certain articles could have been missed. Second, only articles written in English and peer reviewed were included. Hence, preprints and gray literature were left out, which may have introduced some biases. Third, a meta-analysis was not possible due to the heterogeneity of the included studies. Fourth, the quality assessment performed using the MMAT suggests that even if much of the research in this area is of high quality, methodological concerns are significant issues for many studies.

### Conclusions

VR has been applied frequently during the COVID-19 pandemic in medicine, with positive effects for treating several health conditions and for medical education and training. Some barriers need to be overcome for a broader adoption of VR in the health care panorama. Based on these findings, it is possible to offer certain VR-based programs even remotely for therapeutic or educational purposes, and not only VR environments developed ad hoc, but also commercial content can be helpful in clinical or educational support. Moreover, VR-based interventions have the potential to be used effectively for the treatment of several mental and physical conditions, as well as for medical education and training in both immersive and desktop systems. Various VR contents are helpful in treating health problems and for medical education and training, including 360° videos and photos, VR games, and embodied virtual agents.

## Appendix

Multimedia Appendix 1 Mixed Methods Appraisal Tool evaluation.

Multimedia Appendix 2 Study characteristics.

Multimedia Appendix 3 Virtual reality characteristics.

## Abbreviations

HMD: head-mounted display
ICT: information and communication technology
ICU: intensive care unit
MMAT: Mixed Methods Appraisal Tool
RCT: randomized controlled trial
VR: virtual reality

## References

[1] Cucinotta D, Vanelli M (2020). WHO Declares COVID-19 a Pandemic. Acta Biomed.

[2] Di Renzo L, Gualtieri P, Pivari F, Soldati L, Attinà A, Cinelli G, Leggeri C, Caparello G, Barrea L, Scerbo F, Esposito E, De Lorenzo A (2020). Eating habits and lifestyle changes during COVID-19 lockdown: an Italian survey. J Transl Med.

[3] Giuntella O, Hyde K, Saccardo S, Sadoff S (2021). Lifestyle and mental health disruptions during COVID-19. Proc Natl Acad Sci U S A.

[4] Park K, Kim A, Yang M, Lim S, Park J (2021). Impact of the COVID-19 pandemic on the lifestyle, mental health, and quality of life of adults in South Korea. PLoS One.

[5] Kaye A, Okeagu C, Pham A, Silva R, Hurley J, Arron B, Sarfraz N, Lee H, Ghali G, Gamble J, Liu H, Urman R, Cornett E (2021). Economic impact of COVID-19 pandemic on healthcare facilities and systems: International perspectives. Best Pract Res Clin Anaesthesiol.

[6] Chudasama YV, Gillies CL, Zaccardi F, Coles B, Davies MJ, Seidu S, Khunti K (2020). Impact of COVID-19 on routine care for chronic diseases: A global survey of views from healthcare professionals. Diabetes Metab Syndr.

[7] Marasco G, Nardone O, Maida M, Boskoski I, Pastorelli L, Scaldaferri F, Italian Association of Young Gastroenterologist and Endoscopist (AGGEI) (2020). Impact of COVID-19 outbreak on clinical practice and training of young gastroenterologists: A European survey. Dig Liver Dis.

[8] Hall AK, Nousiainen MT, Campisi P, Dagnone JD, Frank JR, Kroeker KI, Brzezina S, Purdy E, Oswald A (2020). Training disrupted: Practical tips for supporting competency-based medical education during the COVID-19 pandemic. Med Teach.

[9] Ahmed H, Allaf M, Elghazaly H (2020). COVID-19 and medical education. Lancet Infect Dis.

[10] Rose S (2020). Medical Student Education in the Time of COVID-19. JAMA.

[11] Pimentel D, Foxman M, Davis DZ, Markowitz DM (2021). Virtually Real, But Not Quite There: Social and Economic Barriers to Meeting Virtual Reality’s True Potential for Mental Health. Front. Virtual Real.

[12] Matamala-Gomez M, Bottiroli S, Realdon O, Riva G, Galvagni L, Platz T, Sandrini G, De Icco R, Tassorelli C (2021). Telemedicine and Virtual Reality at Time of COVID-19 Pandemic: An Overview for Future Perspectives in Neurorehabilitation. Front Neurol.

[13] Woolliscroft JO (2020). Innovation in Response to the COVID-19 Pandemic Crisis. Acad Med.

[14] Asadzadeh A, Samad-Soltani T, Rezaei-Hachesu P (2021). Applications of virtual and augmented reality in infectious disease epidemics with a focus on the COVID-19 outbreak. Inform Med Unlocked.

[15] Imperatori C, Dakanalis A, Farina B, Pallavicini F, Colmegna F, Mantovani F, Clerici M (2020). Global Storm of Stress-Related Psychopathological Symptoms: A Brief Overview on the Usefulness of Virtual Reality in Facing the Mental Health Impact of COVID-19. Cyberpsychol Behav Soc Netw.

[16] Pallavicini F, Chicchi Giglioli Ia, Kim G, Alcañiz M, Rizzo A (2021). Editorial: Virtual Reality, Augmented Reality and Video Games for Addressing the Impact of COVID-19 on Mental Health. Front. Virtual Real.

[17] Singh RP, Javaid M, Kataria R, Tyagi M, Haleem A, Suman R (2020). Significant applications of virtual reality for COVID-19 pandemic. Diabetes Metab Syndr.

[18] Miller HL, Bugnariu NL (2016). Level of Immersion in Virtual Environments Impacts the Ability to Assess and Teach Social Skills in Autism Spectrum Disorder. Cyberpsychol Behav Soc Netw.

[19] Kardong-Edgren S(, Farra SL, Alinier G, Young HM (2019). A Call to Unify Definitions of Virtual Reality. Clinical Simulation in Nursing.

[20] Rebelo F, Noriega P, Duarte E, Soares M (2012). Using virtual reality to assess user experience. Hum Factors.

[21] Parsons TD (2015). Virtual Reality for Enhanced Ecological Validity and Experimental Control in the Clinical, Affective and Social Neurosciences. Front Hum Neurosci.

[22] Izard SG, Juanes JA, García Peñalvo FJ, Estella JMG, Ledesma MJS, Ruisoto P (2018). Virtual Reality as an Educational and Training Tool for Medicine. J Med Syst.

[23] Riva G, Wiederhold BK, Mantovani F (2019). Neuroscience of Virtual Reality: From Virtual Exposure to Embodied Medicine. Cyberpsychol Behav Soc Netw.

[24] Riener R, Harders M (2012). Virtual Reality in Medicine.

[25] Li L, Yu F, Shi D, Shi J, Tian Z, Yang J, Wang X, Jiang Q (2017). Application of virtual reality technology in clinical medicine. Am J Transl Res.

[26] Kyaw BM, Saxena N, Posadzki P, Vseteckova J, Nikolaou CK, George PP, Divakar U, Masiello I, Kononowicz AA, Zary N, Tudor Car L (2019). Virtual Reality for Health Professions Education: Systematic Review and Meta-Analysis by the Digital Health Education Collaboration. J Med Internet Res.

[27] Hilty DM, Randhawa K, Maheu MM, McKean AJS, Pantera R, Mishkind MC, Rizzo A (2020). A Review of Telepresence, Virtual Reality, and Augmented Reality Applied to Clinical Care. J Technol Behav Sci.

[28] Dascal J, Reid M, IsHak WW, Spiegel B, Recacho J, Rosen B, Danovitch I (2017). Virtual Reality and Medical Inpatients: A Systematic Review of Randomized, Controlled Trials. Innov Clin Neurosci.

[29] Freeman D, Reeve S, Robinson A, Ehlers A, Clark D, Spanlang B, Slater M (2017). Virtual reality in the assessment, understanding, and treatment of mental health disorders. Psychol Med.

[30] Kim S, Kim E (2020). The Use of Virtual Reality in Psychiatry: A Review. Soa Chongsonyon Chongsin Uihak.

[31] Maples-Keller JL, Bunnell BE, Kim S, Rothbaum BO (2017). The Use of Virtual Reality Technology in the Treatment of Anxiety and Other Psychiatric Disorders. Harv Rev Psychiatry.

[32] Oing T, Prescott J (2018). Implementations of Virtual Reality for Anxiety-Related Disorders: Systematic Review. JMIR Serious Games.

[33] Wechsler TF, Kümpers F, Mühlberger A (2019). Inferiority or Even Superiority of Virtual Reality Exposure Therapy in Phobias?-A Systematic Review and Quantitative Meta-Analysis on Randomized Controlled Trials Specifically Comparing the Efficacy of Virtual Reality Exposure to Gold Standard Exposure in Agoraphobia, Specific Phobia, and Social Phobia. Front Psychol.

[34] Fodor LA, Coteț CD, Cuijpers P, Szamoskozi Ș, David D, Cristea IA (2018). The effectiveness of virtual reality based interventions for symptoms of anxiety and depression: A meta-analysis. Sci Rep.

[35] Dulau E, Botha-Ravyse C, Luimula M (2019). Virtual reality for physical rehabilitation: A Pilot study How will virtual reality change physical therapy?.

[36] Cho GH, Hwangbo G, Shin HS (2014). The Effects of Virtual Reality-based Balance Training on Balance of the Elderly. J Phys Ther Sci.

[37] Ahmadpour N, Randall H, Choksi H, Gao A, Vaughan C, Poronnik P (2019). Virtual Reality interventions for acute and chronic pain management. Int J Biochem Cell Biol.

[38] Smith V, Warty RR, Sursas JA, Payne O, Nair A, Krishnan S, da Silva Costa F, Wallace EM, Vollenhoven B (2020). The Effectiveness of Virtual Reality in Managing Acute Pain and Anxiety for Medical Inpatients: Systematic Review. J Med Internet Res.

[39] Li A, Montaño Z, Chen VJ, Gold JI (2011). Virtual reality and pain management: current trends and future directions. Pain Manag.

[40] Hoffman HG, Patterson DR, Carrougher GJ (2000). Use of virtual reality for adjunctive treatment of adult burn pain during physical therapy: a controlled study. Clin J Pain.

[41] Stepan K, Zeiger J, Hanchuk S, Del Signore A, Shrivastava R, Govindaraj S, Iloreta A (2017). Immersive virtual reality as a teaching tool for neuroanatomy. Int Forum Allergy Rhinol.

[42] Taubert M, Webber L, Hamilton T, Carr M, Harvey M (2019). Virtual reality videos used in undergraduate palliative and oncology medical teaching: results of a pilot study. BMJ Support Palliat Care.

[43] Barteit S, Lanfermann L, Bärnighausen T, Neuhann F, Beiersmann C (2021). Augmented, Mixed, and Virtual Reality-Based Head-Mounted Devices for Medical Education: Systematic Review. JMIR Serious Games.

[44] Erolin C, Reid L, McDougall S (2019). Using virtual reality to complement and enhance anatomy education. J Vis Commun Med.

[45] Maresky HS, Oikonomou A, Ali I, Ditkofsky N, Pakkal M, Ballyk B (2019). Virtual reality and cardiac anatomy: Exploring immersive three-dimensional cardiac imaging, a pilot study in undergraduate medical anatomy education. Clin Anat.

[46] Zhao J, Xu X, Jiang H, Ding Y (2020). The effectiveness of virtual reality-based technology on anatomy teaching: a meta-analysis of randomized controlled studies. BMC Med Educ.

[47] Hardcastle T, Wood A (2018). The utility of virtual reality surgical simulation in the undergraduate otorhinolaryngology curriculum. J. Laryngol. Otol.

[48] De Ponti R, Marazzato J, Maresca A, Rovera F, Carcano G, Ferrario M (2020). Pre-graduation medical training including virtual reality during COVID-19 pandemic: a report on students' perception. BMC Med Educ.

[49] Kolbe L, Jaywant A, Gupta A, Vanderlind WM, Jabbour G (2021). Use of virtual reality in the inpatient rehabilitation of COVID-19 patients. Gen Hosp Psychiatry.

[50] Atli K, Selman W, Ray A (2021). A Comprehensive Multicomponent Neurosurgical Course with use of Virtual Reality: Modernizing the Medical Classroom. J Surg Educ.

[51] Moher D, Liberati A, Tetzlaff J, Altman DG, PRISMA Group (2009). Preferred reporting items for systematic reviews and meta-analyses: the PRISMA statement. PLoS Med.

[52] Hong QN, Fàbregues S, Bartlett G, Boardman F, Cargo M, Dagenais P, Gagnon M, Griffiths F, Nicolau B, O’Cathain A, Rousseau M, Vedel I, Pluye P (2018). The Mixed Methods Appraisal Tool (MMAT) version 2018 for information professionals and researchers. EFI.

[53] Hong QN, Gonzalez-Reyes A, Pluye P (2018). Improving the usefulness of a tool for appraising the quality of qualitative, quantitative and mixed methods studies, the Mixed Methods Appraisal Tool (MMAT). J Eval Clin Pract.

[54] Cohen J (2016). A Coefficient of Agreement for Nominal Scales. Educational and Psychological Measurement.

[55] Petry NM (2002). A comparison of young, middle-aged, and older adult treatment-seeking pathological gamblers. Gerontologist.

[56] Pigozzi LB, Pereira DD, Pattussi MP, Moret-Tatay C, Irigaray TQ, Weber JBB, Grossi PK, Grossi ML (2021). Quality of life in young and middle age adult temporomandibular disorders patients and asymptomatic subjects: a systematic review and meta-analysis. Health Qual Life Outcomes.

[57] Jaworska N, MacQueen G (2015). Adolescence as a unique developmental period. J Psychiatry Neurosci.

[58] Slater M, Linakis V, Usoh M, Kooper R (1996). Immersion, presence and performance in virtual environments: an experiment with tri-dimensional chess. VRST '96: Proceedings of the ACM Symposium on Virtual Reality Software and Technology.

[59] Schultheis MT, Rizzo AA (2001). The application of virtual reality technology in rehabilitation. Rehabilitation Psychology.

[60] Lampropoulos G, Barkoukis V, Burden K, Anastasiadis T (2021). 360-degree video in education: An overview and a comparative social media data analysis of the last decade. Smart Learn. Environ.

[61] Riva G, Serino S (2020). Virtual Reality in the Assessment, Understanding and Treatment of Mental Health Disorders. J Clin Med.

[62] Pallavicini F, Pepe A, Minissi M (2019). Gaming in Virtual Reality: What Changes in Terms of Usability, Emotional Response and Sense of Presence Compared to Non-Immersive Video Games?. Simulation & Gaming.

[63] Colder Carras M, Van Rooij AJ, Spruijt-Metz D, Kvedar J, Griffiths MD, Carabas Y, Labrique A (2017). Commercial Video Games As Therapy: A New Research Agenda to Unlock the Potential of a Global Pastime. Front Psychiatry.

[64] Zyda M (2005). From visual simulation to virtual reality to games. Computer.

[65] Maloney D, Freeman G (2020). Falling Asleep Together: What Makes Activities in Social Virtual Reality Meaningful to Users. CHI PLAY '20: Proceedings of the Annual Symposium on Computer-Human Interaction in Play.

[66] Sykownik P, Graf L, Zils C, Masuch M (2021).

[67] Landis JR, Koch GG (1977). The measurement of observer agreement for categorical data. Biometrics.

[68] Birrenbach T, Zbinden J, Papagiannakis G, Exadaktylos AK, Müller M, Hautz WE, Sauter TC (2021). Effectiveness and Utility of Virtual Reality Simulation as an Educational Tool for Safe Performance of COVID-19 Diagnostics: Prospective, Randomized Pilot Trial. JMIR Serious Games.

[69] Mottelson A, Vandeweerdt C, Atchapero M, Luong T, Holz C, Böhm R, Makransky G (2021). A self-administered virtual reality intervention increases COVID-19 vaccination intention. Vaccine.

[70] Garcia LM, Birckhead BJ, Krishnamurthy P, Sackman J, Mackey IG, Louis RG, Salmasi V, Maddox T, Darnall BD (2021). An 8-Week Self-Administered At-Home Behavioral Skills-Based Virtual Reality Program for Chronic Low Back Pain: Double-Blind, Randomized, Placebo-Controlled Trial Conducted During COVID-19. J Med Internet Res.

[71] Vlake JH, van Bommel J, Wils E, Bienvenu J, Hellemons ME, Korevaar TI, Schut AF, Labout JA, Schreuder LL, van Bavel MP, Gommers D, van Genderen ME (2022). Intensive Care Unit-Specific Virtual Reality for Critically Ill Patients With COVID-19: Multicenter Randomized Controlled Trial. J Med Internet Res.

[72] Liu Y, Butzlaff A (2021). Where's the germs? The effects of using virtual reality on nursing students' hospital infection prevention during the COVID-19 pandemic. J Comput Assist Learn.

[73] Cecil J, Kauffman S, Gupta A, McKinney V, Miguel Pirela-Cruz MD (2021). Design of a Human Centered Computing (HCC) based Virtual Reality Simulator to train First Responders Involved in the COVID-19 Pandemic.

[74] Riva G, Bernardelli L, Castelnuovo G, Di Lernia D, Tuena C, Clementi A, Pedroli E, Malighetti C, Sforza F, Wiederhold B, Serino S (2021). A Virtual Reality-Based Self-Help Intervention for Dealing with the Psychological Distress Associated with the COVID-19 Lockdown: An Effectiveness Study with a Two-Week Follow-Up. Int J Environ Res Public Health.

[75] Zhang D, Liao H, Jia Y, Yang W, He P, Wang D, Chen Y, Yang W, Zhang Y (2021). Effect of virtual reality simulation training on the response capability of public health emergency reserve nurses in China: a quasiexperimental study. BMJ Open.

[76] Xing Y, Liang Z, Fahy C, Shell J, Guan K, Liu Y, Zhang Q (2021). Virtual Reality Research: Design Virtual Education System for Epidemic (COVID-19) Knowledge to Public. Applied Sciences.

[77] Jeong Y, Lee H, Han J (2022). Development and evaluation of virtual reality simulation education based on coronavirus disease 2019 scenario for nursing students: A pilot study. Nurs Open.

[78] Kang K, Kim S, Lee M, Kim M, Kim S (2020). Comparison of Learning Effects of Virtual Reality Simulation on Nursing Students Caring for Children with Asthma. Int J Environ Res Public Health.

[79] da Silva TD, da Silva PL, Valenzuela EDJ, Dias ED, Simcsik AO, de Carvalho MG, Fontes AMGG, Alberissi CADO, de Araújo LV, Brandão MVC, Dawes H, Monteiro CBDM (2021). Serious Game Platform as a Possibility for Home-Based Telerehabilitation for Individuals With Cerebral Palsy During COVID-19 Quarantine - A Cross-Sectional Pilot Study. Front Psychol.

[80] Beverly E, Hommema L, Coates K, Duncan G, Gable B, Gutman T, Love M, Love C, Pershing M, Stevens N (2022). A tranquil virtual reality experience to reduce subjective stress among COVID-19 frontline healthcare workers. PLoS One.

[81] Nijland JWHM, Veling W, Lestestuiver BP, Van Driel CMG (2021). Virtual Reality Relaxation for Reducing Perceived Stress of Intensive Care Nurses During the COVID-19 Pandemic. Front Psychol.

[82] Sadeghi AH, Wahadat AR, Dereci A, Budde RPJ, Tanis W, Roos-Hesselink JW, Takkenberg H, Taverne YJHJ, Mahtab EAF, Bogers AJJC (2021). Remote multidisciplinary heart team meetings in immersive virtual reality: a first experience during the COVID-19 pandemic. BMJ Innov.

[83] Guichet PL, Huang J, Zhan C, Millet A, Kulkarni K, Chhor C, Mercado C, Fefferman N (2022). Incorporation of a Social Virtual Reality Platform into the Residency Recruitment Season. Acad Radiol.

[84] Leung LY, Malthouse T, Chong J, Down C, Crawford R, Coker C, Dhanda J (2021). PD02-11 Utilising 360 Live Streaming to Deliver Immersive Urology Education in the COVID-19 Era with Virtual Reality in Medicine and Surgery (VRIMS). Journal of Urology.

[85] Zhang W, Paudel D, Shi R, Liang J, Liu J, Zeng X, Zhou Y, Zhang B (2020). Virtual Reality Exposure Therapy (VRET) for Anxiety Due to Fear of COVID-19 Infection: A Case Series. Neuropsychiatr Dis Treat.

[86] Siani A, Marley SA (2021). Impact of the recreational use of virtual reality on physical and mental wellbeing during the Covid-19 lockdown. Health Technol (Berl).

[87] Speidel R, Schneider A, Körner J, Grab-Kroll C, Öchsner W (2021). Did video kill the XR star? Digital trends in medical education before and after the COVID-19 outbreak from the perspective of students and lecturers from the faculty of medicine at the University of Ulm. GMS J Med Educ.

[88] Wagner M, Jaki C, Löllgen RM, Mileder L, Eibensteiner F, Ritschl V, Steinbauer P, Gottstein M, Abulebda K, Calhoun A, Gross IT (2021). Readiness for and Response to Coronavirus Disease 2019 Among Pediatric Healthcare Providers: The Role of Simulation for Pandemics and Other Disasters. Pediatr Crit Care Med.

[89] Tsekhmister YV, Konovalova T, Tsekhmister BY, Agrawal A, Ghosh D (2021). Evaluation of Virtual Reality Technology and Online Teaching System for Medical Students in Ukraine During COVID-19 Pandemic. Int. J. Emerg. Technol. Learn.

[90] Campo-Prieto P, Rodríguez-Fuentes G, Cancela-Carral JM (2021). Immersive Virtual Reality Exergame Promotes the Practice of Physical Activity in Older People: An Opportunity during COVID-19. MTI.

[91] Yahara M, Niki K, Ueno K, Okamoto M, Okuda T, Tanaka H, Naito Y, Ishii R, Ueda M, Ito T (2021). Remote Reminiscence Using Immersive Virtual Reality May Be Efficacious for Reducing Anxiety in Patients with Mild Cognitive Impairment Even in COVID-19 Pandemic: A Case Report. Biol Pharm Bull.

[92] Yang T, Lai IKW, Fan ZB, Mo QM (2021). The impact of a 360° virtual tour on the reduction of psychological stress caused by COVID-19. Technol Soc.

[93] Oulefki A, Agaian S, Trongtirakul T, Benbelkacem S, Aouam D, Zenati-Henda N, Abdelli M (2022). Virtual Reality visualization for computerized COVID-19 lesion segmentation and interpretation. Biomed Signal Process Control.

[94] Yang J, Menhas R, Dai J, Younas T, Anwar U, Iqbal W, Ahmed Laar R, Muddasar Saeed M (2022). Virtual Reality Fitness (VRF) for Behavior Management During the COVID-19 Pandemic: A Mediation Analysis Approach. PRBM.

[95] Paul M, Bullock K, Bailenson J (2020). Virtual Reality Behavioral Activation as an Intervention for Major Depressive Disorder: Case Report. JMIR Ment Health.

[96] Vlake JH, van Bommel J, Hellemons ME, Wils E, Gommers D, van Genderen ME (2020). Intensive Care Unit-Specific Virtual Reality for Psychological Recovery After ICU Treatment for COVID-19; A Brief Case Report. Front Med (Lausanne).

[97] Sampaio M, Navarro Haro MV, Wilks C, De Sousa B, Garcia-Palacios A, Hoffman H (2021). Spanish-Speaking Therapists Increasingly Switch to Telepsychology During COVID-19: Networked Virtual Reality May Be Next. Telemed J E Health.

[98] Sampaio M, Haro MVN, De Sousa B, Melo WV, Hoffman HG (2021). Therapists Make the Switch to Telepsychology to Safely Continue Treating Their Patients During the COVID-19 Pandemic. Virtual Reality Telepsychology May Be Next. Front Virtual Real.

[99] Ralston B, Willett R, Namperumal S, Brown N, Walsh H, Muñoz RA, Del Castillo S, Chang T, Yurasek G (2021). Use of Virtual Reality for Pediatric Cardiac Critical Care Simulation. Cureus.

[100] Buyego P, Katwesigye E, Kebirungi G, Nsubuga M, Nakyejwe S, Cruz P, McCarthy M, Hurt D, Kambugu A, Arinaitwe JW, Ssekabira U, Jjingo D (2021). Feasibility of Virtual Reality based Training for Optimising COVID-19 Case Handling in Uganda. Res Sq.

[101] Herbst R, Rybak T, Meisman A, Whitehead M, Rosen B, Crosby LE, Klein MD, Real FJ (2021). A Virtual Reality Resident Training Curriculum on Behavioral Health Anticipatory Guidance: Development and Usability Study. JMIR Pediatr Parent.

[102] Petrica A, Lungeanu D, Ciuta A, Marza AM, Botea M, Mederle OA (2021). Using 360-degree video for teaching emergency medicine during and beyond the COVID-19 pandemic. Ann Med.

[103] Bridge P, Shiner N, Bolderston A, Gunn T, Hazell L, Johnson R, Lawson Jones G, Mifsud L, Stewart S, McNulty J (2021). International audit of simulation use in pre-registration medical radiation science training. Radiography (Lond).

[104] Flo J, Byermoen KR, Egilsdottir HÖ, Eide H, Heyn LG (2021). Nursing students' experiences of virtual simulation when using a video conferencing system - a mixed methods study. Int J Nurs Educ Scholarsh.

[105] Barreda-Ángeles M, Hartmann T (2022). Psychological benefits of using social virtual reality platforms during the covid-19 pandemic: The role of social and spatial presence. Comput Human Behav.

[106] Anderson-Hanley C, Maloney M, Barcelos N, Striegnitz K, Kramer A (2017). Neuropsychological Benefits of Neuro-Exergaming for Older Adults: A Pilot Study of an Interactive Physical and Cognitive Exercise System (iPACES). J Aging Phys Act.

[107] Lindner P, Miloff A, Hamilton W, Carlbring P (2019). The Potential of Consumer-Targeted Virtual Reality Relaxation Applications: Descriptive Usage, Uptake and Application Performance Statistics for a First-Generation Application. Front Psychol.

[108] Pallavicini F, Pepe A (2020). Virtual Reality Games and the Role of Body Involvement in Enhancing Positive Emotions and Decreasing Anxiety: Within-Subjects Pilot Study. JMIR Serious Games.

[109] Indovina P, Barone D, Gallo L, Chirico A, De Pietro G, Giordano A (2018). Virtual Reality as a Distraction Intervention to Relieve Pain and Distress During Medical Procedures: A Comprehensive Literature Review. Clin J Pain.

[110] Spiegel B, Fuller G, Lopez M, Dupuy T, Noah B, Howard A, Albert M, Tashjian V, Lam R, Ahn J, Dailey F, Rosen BT, Vrahas M, Little M, Garlich J, Dzubur E, IsHak W, Danovitch I (2019). Virtual reality for management of pain in hospitalized patients: A randomized comparative effectiveness trial. PLoS One.

[111] Qian J, McDonough DJ, Gao Z (2020). The Effectiveness of Virtual Reality Exercise on Individual's Physiological, Psychological and Rehabilitative Outcomes: A Systematic Review. Int J Environ Res Public Health.

[112] Pallavicini F, Pepe A (2019). Comparing Player Experience in Video Games Played in Virtual Reality or on Desktop Displays: Immersion, Flow, and Positive Emotions.

[113] Li J, Erdt M, Chen L, Cao Y, Lee S, Theng Y (2018). The Social Effects of Exergames on Older Adults: Systematic Review and Metric Analysis. J Med Internet Res.

[114] Marques A, Peralta M, Martins J, Catunda R, Matos MGD, Saboga Nunes L (2016). Associations between physical activity and self-rated wellbeing in European adults: A population-based, cross-sectional study. Prev Med.

[115] Birckhead B, Eberlein S, Alvarez G, Gale R, Dupuy T, Makaroff K, Fuller G, Liu X, Yu K, Black JT, Ishimori M, Venuturupalli S, Tu J, Norris T, Tighiouart M, Ross L, McKelvey K, Vrahas M, Danovitch I, Spiegel B (2021). Home-based virtual reality for chronic pain: protocol for an NIH-supported randomised-controlled trial. BMJ Open.

[116] Pedram S, Palmisano S, Perez P, Mursic R, Farrelly M (2020). Examining the potential of virtual reality to deliver remote rehabilitation. Computers in Human Behavior.

[117] Pallavicini F, Orena E, di Santo S, Greci L, Caragnano C, Ranieri P, Vuolato C, Pepe A, Veronese G, Stefanini S, Achille F, Dakanalis A, Bernardelli L, Sforza F, Rossini A, Caltagirone C, Fascendini S, Clerici M, Riva G, Mantovani F (2022). A virtual reality home-based training for the management of stress and anxiety among healthcare workers during the COVID-19 pandemic: study protocol for a randomized controlled trial. Trials.

[118] Pallavicini F, Pepe A, Mantovani F (2021). Commercial Off-The-Shelf Video Games for Reducing Stress and Anxiety: Systematic Review. JMIR Ment Health.

[119] Parong J, Mayer RE (2018). Learning science in immersive virtual reality. Journal of Educational Psychology.

[120] Webster R (2015). Declarative knowledge acquisition in immersive virtual learning environments. Interactive Learning Environments.

[121] De Freitas FS, Rebolledo-Mendez G, Liarokapis F, Magoulas G, Poulovassilis A (2010). Learning as immersive experiences: Using the four-dimensional framework for designing and evaluating immersive learning experiences in a virtual world. British Journal of Educational Technology.

[122] Pottle J (2019). Virtual reality and the transformation of medical education. Future Healthc J.

[123] Triandafilou KM, Tsoupikova D, Barry AJ, Thielbar KN, Stoykov N, Kamper DG (2018). Development of a 3D, networked multi-user virtual reality environment for home therapy after stroke. J Neuroeng Rehabil.

[124] Schild J, Misztal S, Roth B, Flock L, Luiz T, Lerner D, Herkersdorf M, Weaner K, Neuberaer M, Franke A, Kemp C, Pranqhofer J, Seele S, Buhler H, Herpers R (2018). Applying Multi-User Virtual Reality to Collaborative Medical Training.

[125] Riva G, Wiederhold BK (2022). What the Metaverse Is (Really) and Why We Need to Know About It. Cyberpsychol Behav Soc Netw.

[126] Asiedu KG Meta’s next big bet: The ‘metaversity’. Protocol.

[127] Jimenez YA, Cumming S, Wang W, Stuart K, Thwaites DI, Lewis SJ (2018). Patient education using virtual reality increases knowledge and positive experience for breast cancer patients undergoing radiation therapy. Support Care Cancer.

[128] Daricello L, Leonardi L, Maggio A, Orlando S, Pillitteri I, Bocchino F, Daricello L, Leonardi L, Maggio A, Orlando S, Pillitteri I, Bocchino F (2020). Virtual and Augmented Reality for increasing the awareness of current scientific research.

[129] Palanica A, Docktor MJ, Lee A, Fossat Y (2019). Using mobile virtual reality to enhance medical comprehension and satisfaction in patients and their families. Perspect Med Educ.

[130] Pandrangi VC, Gaston B, Appelbaum NP, Albuquerque FC, Levy MM, Larson RA (2019). The Application of Virtual Reality in Patient Education. Ann Vasc Surg.

[131] Chang S, Kuo M, Lin Y, Chen S, Chen C, Yang Y, Yang L, Kao S, Shulruf B, Lee F (2021). Virtual reality-based preprocedural education increases preparedness and satisfaction of patients about the catheter ablation of atrial fibrillation. J Chin Med Assoc.

[132] Jerdan SW, Grindle M, van Woerden HC, Kamel Boulos MN (2018). Head-Mounted Virtual Reality and Mental Health: Critical Review of Current Research. JMIR Serious Games.

[133] Concannon BJ, Esmail S, Roduta Roberts M (2019). Head-Mounted Display Virtual Reality in Post-secondary Education and Skill Training. Front. Educ.

[134] Bevilacqua R, Maranesi E, Riccardi GR, Donna VD, Pelliccioni P, Luzi R, Lattanzio F, Pelliccioni G (2019). Non-Immersive Virtual Reality for Rehabilitation of the Older People: A Systematic Review into Efficacy and Effectiveness. J Clin Med.

[135] Hamilton D, McKechnie J, Edgerton E, Wilson C (2020). Immersive virtual reality as a pedagogical tool in education: a systematic literature review of quantitative learning outcomes and experimental design. J. Comput. Educ.

[136] Gladden M (2018). A Phenomenological Framework of Architectural Paradigms for the User-Centered Design of Virtual Environments. MTI.

[137] Jensen L, Konradsen F (2017). A review of the use of virtual reality head-mounted displays in education and training. Educ Inf Technol.

[138] Cooper N, Milella F, Pinto C, Cant I, White M, Meyer G (2018). The effects of substitute multisensory feedback on task performance and the sense of presence in a virtual reality environment. PLoS One.

[139] Marsh T, Wright P, Smith S (2001). Evaluation for the design of experience in virtual environments: modeling breakdown of interaction and illusion. Cyberpsychol Behav.

[140] Pallavicini F, Cipresso P, Raspelli S, Grassi A, Serino S, Vigna C, Triberti S, Villamira M, Gaggioli A, Riva G (2013). Is virtual reality always an effective stressors for exposure treatments? Some insights from a controlled trial. BMC Psychiatry.

[141] Lindner P, Hamilton W, Miloff A, Carlbring P (2019). How to Treat Depression With Low-Intensity Virtual Reality Interventions: Perspectives on Translating Cognitive Behavioral Techniques Into the Virtual Reality Modality and How to Make Anti-Depressive Use of Virtual Reality-Unique Experiences. Front Psychiatry.

[142] Gabbard J, Hix D, Swan J (1999). User-centered design and evaluation of virtual environments. IEEE Comput. Grap. Appl.

[143] Pizzoli SFM, Mazzocco K, Triberti S, Monzani D, Alcañiz Raya ML, Pravettoni G (2019). User-Centered Virtual Reality for Promoting Relaxation: An Innovative Approach. Front Psychol.

[144] Pallavicini F, Orena E, di Santo S, Greci L, Caragnano C, Ranieri P, Vuolato C, Pepe A, Veronese G, Dakanalis A, Rossini A, Caltagirone C, Clerici M, Mantovani F (2021). MIND-VR: Design and Evaluation Protocol of a Virtual Reality Psychoeducational Experience on Stress and Anxiety for the Psychological Support of Healthcare Workers Involved in the COVID-19 Pandemic. Front. Virtual Real.

[145] Angelov V, Petkov E, Shipkovenski G, Kalushkov T (2020). Modern Virtual Reality Headsets.

