# Supplementary material for: Virtual Reality Applications in Medicine During the COVID-19 Pandemic: Systematic Review
Source: JMIR Serious Games. 2022 Oct 25;10(4):e35000. doi: 10.2196/35000 (PMC9605086; doi:10.2196/35000)
Supplement: Multimedia Appendix 1 [file games_v10i4e35000_app1.docx]

**Multimedia Appendix 1. Mixed Methods Appraisal Tool evaluation.**

|  | **2. Quantitative randomized controlled trial** | | | | | **3. Quantitative  non-randomized** | | | | | **4. Quantitative**  **descriptive** | | | | | **5. Mixed-methods** | | | | | **Overall score** |
| --- | --- | --- | --- | --- | --- | --- | --- | --- | --- | --- | --- | --- | --- | --- | --- | --- | --- | --- | --- | --- | --- |
| **Study** | **2.1** | **2.2** | **2.3** | **2.4** | **2.5** | **3.1** | **3.2** | **3.3** | **3.4** | **3.5** | **4.1** | **4.2** | **4.3** | **4.4** | **4.5** | **5.1** | **5.2** | **5.3** | **5.4** | **5.5** |  |
| Beverly et al. (2022) [80] |  |  |  |  |  | Yes | Yes | Yes | No | Yes |  |  |  |  |  |  |  |  |  |  | **** |
| Birrebach et al. (2021) [68] | No | Yes | Yes | Can’t tell | Yes |  |  |  |  |  |  |  |  |  |  |  |  |  |  |  | *** |
| Bridge et al. (2021) [103] |  |  |  |  |  |  |  |  |  |  |  |  |  |  |  | Yes | Yes | Yes | Yes | Yes | ***** |
| Buyego et al. (2021) [100] |  |  |  |  |  |  |  |  |  |  |  |  |  |  |  | Yes | Yes | Yes | No | No | *** |
| Campo-Preito et al. (2021) [90] |  |  |  |  |  |  |  |  |  |  | Yes | Yes | Yes | No | Yes |  |  |  |  |  | **** |
| Cecil et al. (2021) [73] |  |  |  |  |  | No | Yes | Yes | No | Yes |  |  |  |  |  |  |  |  |  |  | *** |
| De Ponti et al. (2020) [48] |  |  |  |  |  |  |  |  |  |  | Yes | No | No | Yes | No |  |  |  |  |  | ** |
| Flo et al. (2021) [104] |  |  |  |  |  |  |  |  |  |  |  |  |  |  |  | Yes | Yes | Yes | Yes | Yes | ***** |
| Garcia et al. (2021) [70] | Yes | Yes | Yes | Yes | Yes |  |  |  |  |  |  |  |  |  |  |  |  |  |  |  | ***** |
| Guichet et al. (2021) [83] |  |  |  |  |  |  |  |  |  |  | Yes | No | No | Yes | No |  |  |  |  |  | ** |
| Herbst et al. (2021) [101] |  |  |  |  |  |  |  |  |  |  |  |  |  |  |  | Yes | Yes | Yes | Yes | Yes | ***** |
| Jeong et al (2022) [77] |  |  |  |  |  | Yes | Yes | Yes | No | Yes |  |  |  |  |  |  |  |  |  |  | **** |
| Kang et al. (2020) [78] |  |  |  |  |  | Yes | Yes | Yes | No | Yes |  |  |  |  |  |  |  |  |  |  | **** |
| Kolbe et al. (2021) [49] |  |  |  |  |  |  |  |  |  |  | Yes | Yes | No | No | Yes |  |  |  |  |  | *** |
| Leung et al. (2021) [84] |  |  |  |  |  |  |  |  |  |  | No | Yes | No | No | Yes |  |  |  |  |  | ** |
| Liu & Butzlaff (2021) [72] |  |  |  |  |  | No | No | Yes | No | Yes |  |  |  |  |  |  |  |  |  |  | ** |
| Mottelson et al. (2021) [69] | No | No | Yes | Yes | Yes |  |  |  |  |  |  |  |  |  |  |  |  |  |  |  | *** |
| Nijland et al. (2021) [81] |  |  |  |  |  | Yes | Yes | Yes | No | Yes |  |  |  |  |  |  |  |  |  |  | **** |
| Oulefki et al. (2022) [93] |  |  |  |  |  |  |  |  |  |  | Yes | No | Yes | Yes | Yes |  |  |  |  |  | **** |
| Paul et al. (2020) [95] |  |  |  |  |  |  |  |  |  |  | Yes | Yes | Yes | Yes | Yes |  |  |  |  |  | ***** |
| Petrica et al. (2021) [102] |  |  |  |  |  |  |  |  |  |  |  |  |  |  |  | Yes | Yes | Yes | No | Yes | **** |
| Rastlon et al. (2021) [99] |  |  |  |  |  |  |  |  |  |  |  |  |  |  |  | Yes | No | No | No | No | * |
| Riva et al. (2021) [74] |  |  |  |  |  | Yes | Yes | Yes | No | Yes |  |  |  |  |  |  |  |  |  |  | **** |
| Sadeghi et al. (2021) [82] |  |  |  |  |  |  |  |  |  |  | Yes | Yes | No | No | No |  |  |  |  |  | ** |
| Sampaio et al. (2021) [98] |  |  |  |  |  |  |  |  |  |  | Yes | Yes | Yes | Yes | Yes |  |  |  |  |  | ***** |
| Sampaio et al. (2021) [97] |  |  |  |  |  |  |  |  |  |  | Yes | Yes | Yes | Yes | Yes |  |  |  |  |  | ***** |
| Siani et al. (2021) [86] |  |  |  |  |  |  |  |  |  |  | Yes | Yes | No | No | Yes |  |  |  |  |  | *** |
| Silva et al. (2021) [79] |  |  |  |  |  | Yes | Yes | Yes | No | Yes |  |  |  |  |  |  |  |  |  |  | **** |
| Speidel et al. (2021) [87] |  |  |  |  |  |  |  |  |  |  | Yes | No | Yes | No | Yes |  |  |  |  |  | *** |
| Vlake et al. (2021) [96] |  |  |  |  |  |  |  |  |  |  | Yes | Yes | Yes | Yes | Yes |  |  |  |  |  | ***** |
| Vlake et al. (2022) [71] | Yes | Yes | Yes | Yes | Yes |  |  |  |  |  |  |  |  |  |  |  |  |  |  |  | ***** |
| Volodymyrovych et al. (2021) [89] |  |  |  |  |  |  |  |  |  |  | Yes | No | Yes | No | Yes |  |  |  |  |  | *** |
| Wagner et al. (2021) [88] |  |  |  |  |  |  |  |  |  |  | Yes | No | No | Yes | Yes |  |  |  |  |  | *** |
| Xing et al. (2021) [76] |  |  |  |  |  | No | Yes | Yes | No | Yes |  |  |  |  |  |  |  |  |  |  | *** |
| Yahara et al. (2021) [91] |  |  |  |  |  |  |  |  |  |  | Yes | Yes | Yes | No | Yes |  |  |  |  |  | **** |
| Yang et al. (2021) [92] |  |  |  |  |  |  |  |  |  |  | Yes | Yes | No | Yes | Yes |  |  |  |  |  | **** |
| Yang et al. (2022) [94] |  |  |  |  |  |  |  |  |  |  | Yes | No | Yes | Yes | Yes |  |  |  |  |  | **** |
| Zhang et al. (2021) [75] |  |  |  |  |  | No | Yes | Yes | No | Yes |  |  |  |  |  |  |  |  |  |  | *** |
| Zhang et al. (2020) [85] |  |  |  |  |  |  |  |  |  |  | Yes | No | Yes | No | Yes |  |  |  |  |  | *** |
