# Supplementary material for: Virtual Reality Applications in Medicine During the COVID-19 Pandemic: Systematic Review
Source: JMIR Serious Games. 2022 Oct 25;10(4):e35000. doi: 10.2196/35000 (PMC9605086; doi:10.2196/35000)
Supplement: Multimedia Appendix 2 [file games_v10i4e35000_app2.docx]

**Multimedia Appendix 2. Study characteristics.**

| **Study** | **Area** | **Aim** | **Time and place** | **Sample** | **Study Design** | **Outcome measures** | **Main outcomes** |
| --- | --- | --- | --- | --- | --- | --- | --- |
| Beverly et al. (2022) [80] | Treatment  (mental health) | To create a Tranquil Cine-VR simulation to reduce subjective stress among frontline workers employed at COVID-19 treatment units | During the spring of 2021, in the midwestern US | 102 frontline workers  (71.6% female, N=73; age range 25-34 years old) | Quantitative  non-randomized | Self-reported questionnaires:   - Demographic information - Visual analogue scale (VAS) to rate stress | Findings support the use of the Tranquil Cine-VR simulation as a brief, accessible intervention to reduce subjective stress among frontline healthcare workers in COVID-19 treatment units |
| Birrebach  et al. (2021) [68] | Education and training | To explore the short- and long-term effectiveness of a fully immersive VR simulation versus a traditional learning method regarding a COVID-19–related skillset | From September to November  2020 in Switzerland | 29 medical students (63% female, N=18; mean age= 22.7) | Quantitative randomized controlled trial  Two conditions:   - VR simulation (Experimental Group) - Video-based instruction - (Control Group) | Self-reported questionnaires:   - Demographic information - User Satisfaction Evaluation Questionnaire (USEQ) - Simulator Sickness Questionnaire (SSQ) - Slater-Usoh-Steed questionnaire (SUS) - After-Scenario Questionnaire (ASQ)   Performance task:   - Hand task | VR simulation resulted at least as effective as traditional learning methods in training medical students while providing benefits regarding user satisfaction |
| Bridge et al. (2021) [103] | Education and training | To quantify usage of simulation-based education (SBE) resources and activities internationally and to evaluate how this changed during COVID-19 restrictions | In September 2020 in UK | 72 participants from Medical Radiation Science (MRS) programs internationally (age and gender unspecified) | Mixed methods | Self-reported questionnaires:   - Demographic information - Custom multiple option, matrix and short-answer open questions on simulation activities, future plans and how COVID-19 restrictions had impacted on use of SBE | Where available, high-fidelity VR and specialized profession-specific resources were used heavily |
| Buyego et al. (2021) [100] | Education and training | To explore the use of VR simulations for frontline workers' training since it trains participants without exposing them to infections that would arise from in-person training | During the first year of the COVID-19 pandemic in Uganda | 55 participants recruited among frontline health workers (56.4% female, N=31; age unspecified) | Mixed methods | Self-reported questionnaires:   - Demographic information - Custom quantitative and open-ended questions on the course (e.g., strength, weakness)   Knowledge assessment:   - Comparison of similar outcomes in previous cohorts | The formalized comparison with classroom-trained cohorts showed relatively better outcomes of VR by way of skills acquired, speed of learning and rates of information retention |
| Campo-Preito et al. (2021) [90] | Treatment  (physical activity) | To analyze the use of immersive VR exergames as a tool to facilitate physical exercise in older people | In July 2021 in Spain | 4 healthy older adults (all male, age range  65–77) | Quantitative descriptive (Case report) | Self-reported questionnaires:   - Demographic information - Simulator Sickness Questionnaire (SSQ) - System Usability Scale (SUS) - Game Experience Questionnaire (GEQ) - Ad hoc satisfaction and perceived effort questionnaire | All participants completed the sessions without adverse effects and reported high levels of satisfaction with the VR experience |
| Cecil et al. (2021) [73] | Education and training | To explore the adoption of VR-based training for nurses during the COVID-19 period and to investigate the impact of audio/visual distractions and interruptions on participant comprehension | During the first year of the COVID-19 pandemic in the US | 30 nurses and  nurse trainee experts (age and gender unspecified) | Quantitative  non-randomized  Two conditions:   - Distraction Group - No distraction Group | Self-reported questionnaires:   - Assessment of affordance   Knowledge assessment:   - Questions related to pre-swabbing, swabbing, and post-swabbing tasks   Physiological data:   - Heart rate | VR-based environments provide an effective and safe alternative to training nurses virtually (rather than face-to-face physical training). Audio/visual distractions and interruptions negatively affected the ability of participants to comprehend a target scenario |
| De Ponti et al. (2020) [48] | Education and training | To assess medical students' perception of a remote VR training, including simulated clinical scenarios during the COVID-19 pandemic | From May to July 2020 in Italy | 115 students attending the sixth year of the course of Medicine and Surgery (age and gender unspecified) | Quantitative descriptive (Survey) | Self-reported questionnaires:   - Demographic information - Custom quantitative questions on the perceived quality of medical training received | Medical students positively responded to the perceived quality of this training modality. A non-negligible proportion of students experienced difficulties in online access to this VR platform |
| Flo et al. (2021) [104] | Education and training | To explore how undergraduate nursing students experienced learning through VR during the COVID-19 pandemic | In March 2020 in Norway | 69 nursing students in their second year (93.9% female, N=31; mean age= 24.3) | Mixed methods | Self-reported questionnaires:   - Demographic information - Custom quantitative questions on the students' learning process, the pedagogical process, and students' expectations of the Body Interact™ simulator   Focus group and semi-structured interview:   - Focused on students' experiences   Users’ sessions data:   - System information on use | The virtual simulation helped them to bridge gaps in both the teaching and learning processes |
| Garcia et al. (2021) [70] | Treatment  (distraction) | To investigate the usefulness of a self-administered behavioral skills-based VR program in individuals with self-reported chronic low back pain during the COVID-19 pandemic | In November 2020 in the US | 79 adults suffering from chronic low back pain (76.5% female, N=137; mean age= 51.5, SD =13.1) | Quantitative randomized controlled trial  Two conditions:   - 3D content using the VR headset (Experimental Group) - 2D images using the VR headset (Control Group) | Self-reported questionnaires:   - Demographic information and pain duration - Defense and Veterans Pain Rating Scale (DVPRS) - DVPRS interference scale (DVPRS-II) - Patient’s Global Impression of Change (PGIC) - Physical Function and Sleep Disturbance (PROMIS) - Pain Catastrophizing Scale (PCS) - Pain Self-Efficacy Questionnaire (PSEQ-2) - Chronic Pain Acceptance Questionnaire - (CPAQ-8) - Satisfaction With Treatment - System Usability Scale - Motion Sickness and Nausea (Cybersickness) - Over-the-Counter Analgesic Medication Use and Opioid Use Data | The 3D pain self-management VR program had high user satisfaction and superior and clinically meaningful symptom reduction for average pain intensity and pain-related interference with activity, mood, and stress compared to active control (i.e., 2D content within a VR headset) |
| Guichet et al. (2021) [83] | Education and training | To assess applicants’ attitudes towards a social  VR platform for the pre-interview social during the 2020-2021 radiology residency | In February 2021 in the US | 120 radiology residency applicants (age and gender unspecified) | Quantitative descriptive (Survey) | Self-reported questionnaires:   - Demographic information - Statements to gauge applicants’ attitudes towards the social VR platform and its usefulness in helping them learn about the residency program | Applicants reported that the use of the social VR platform reflected positively on the residency and positively impacted their decision to consider the program strongly |
| Herbst et al. (2021) [101] | Education and training | To explore the usability of a VR-based behavioral health anticipatory guidance curriculum for pediatric  Residents during the COVID-19 pandemic | During the first year of the COVID-19 pandemic in the US | 14 postgraduate third-year pediatric residents (79% female, N=11; mean age= 29.4, SD= 2.6) | Mixed methods | Self-reported questionnaires:   - Demographic information - MEC Spatial Presence Questionnaire   Semi-structured interview:   - Residents’ perspectives on the curriculum’s content and format | Residents experienced VR as immersive, feasible, realistic to the clinic setting, and a safe space to practice and learn new skills |
| Jeong et al (2022)  [77] | Education and training | To develop and assess the effectiveness of a VR-based simulation program for patients with respiratory  infectious diseases based on a COVID-19 scenario for nursing students | From December 2020 to January 2021 in South Korea | 65 participants recruited among fourth-year nursing students (83.1% female, N=54; age unspecified) | Quantitative  non-randomized  Two conditions:   - VR simulation   (Experimental group)   - Video (Control group) | Self-reported questionnaires:   - Demographic information - Quantitative questions on self-efficacy for nursing care of respiratory infectious diseases - Quantitative questions on clinical reasoning capability - Quantitative question on learning satisfaction   Knowledge related to respiratory infectious diseases   - 10 quantitative ad hoc questions | The VR group showed a significantly higher learning satisfaction |
| Kang et al. (2020) [78] | Education and training | To examine the learning effects of VR simulation (vSim) by comparing three different educational modalities of nursing care for children with asthma | From March to July 2020 in South Korea | 192 participants recruited from junior nursing students located in different schools in South Korea (80.2% female, N= 154; mean age= 22.3, SD=1.8) | Quantitative  non-randomized  Three conditions:   - VR simulation (vSim) (Group 1) - High-fidelity simulation (HFS) (Group 2) - Combination of learning methods (vSim with HFS) (Group 3) | Self-reported questionnaires:   - Demographic information - Custom quantitative items on Knowledge scale of nursing care for children with asthma - Custom quantitative items on Confidence in Practice (CP)   Performance test:   - Clinical performance tool | Simulation practice using vSim combined with HFS could be an effective educational method for nursing students |
| Kolbe et al. (2021) [49] | Treatment  (isolation) | To understand patient satisfaction and the perceived benefit of VR on a COVID-19 recovery unit (CRU), as well as the logistical and operational feasibility of providing VR content | During the COVID-19 surge in New York City in 2020 | 24 patients from a COVID-19 rehabilitation unit (age and gender unspecified) | Quantitative descriptive (Survey) | Self-reported questionnaires:   - Demographic information - Custom quantitative questions to understand satisfaction and perceived benefit of VR to patients and staff | The VR program was highly satisfactory, with perceived benefits in coping with isolation and loneliness |
| Leung et al. (2021) [84] | Education and training | To investigate the usefulness of a VR cadaveric course in urology during the COVID-19 pandemic | During the first year of the COVID-19 pandemic in the UK | 15 medical students (age and gender unspecified) | Quantitative descriptive (Survey) | Self-reported questionnaires:   - Demographic information - Custom quantitative questions on the learning experience | The VR course was very or extremely useful, added to their learning experience. Students benefited specifically from observing procedures that they would otherwise not experience |
| Liu & Butzlaff (2021) [72] | Education and training | To examine the effects of incorporating a VR activity into the traditional classroom and in an online environment to enhance nursing education | Before the COVID-19 pandemic (Time 1) and during the COVID-19 pandemic (Time 2) in the US | 50 junior-level nursing students during their first medical-surgical (74% female, N=37; mean age=23, SD=3) | Quantitative  non-randomized  Two conditions:   - VR activity while online (Group 1) - VR activity while in the classroom (Group 2) | Self-reported questionnaires:   - Demographic information - Custom quantitative questions on satisfaction with the learning activity and the web-based tour creator platform   Knowledge assessment:   - Students were asked to identify infectious sites based on their own knowledge | Results showed students’ positive perceptions and performances in both traditional classroom and remote settings |
| Mottelson et al. (2021) [69] | Education and training | To evaluate a VR-based intervention for communicating the benefits of COVID-19 vaccination | From April to May 2021 in Denmark | 282 adults not vaccinated against COVID-19 (7% female, N= 20; mean age= 28.9, SD=9.7) | Quantitative randomized controlled trial  Four conditions:   - Two age conditions (young or old self-body) - Two communication conditions (with provision of personal benefit of vaccination only, or collective and personal benefit) | Self-reported questionnaires:   - Demographic information - Vaccination intention (score range 1–100) measured three times: immediately before and after the study, as well as one week later | The VR intervention was effective in increasing COVID-19 vaccination intentions, with significant retention one week after the intervention |
| Nijland et al. (2021)  [81] | Treatment  (mental health) | To investigate the feasibility  and immediate effect on perceived stress of VR relaxation (VRelax) use by ICU nurses working with COVID-19 patients during work shifts | In May 2020 in the Netherlands | 138 ICU nurses working in intensive care units for COVID-19 patients (84% female, N= 116; mean age= 42, SD=11.7) | Quantitative  non-randomized  Two conditions:   - VR (Experimental group) - Video (Control group) | Self-reported questionnaires:   - Demographic information - Perceived Stress Scale (PSS) - Connor-Davidson Resilience Scale (CD-RISC-10) - Custom questions on VRelax user experiences | VRelax was an effective  intervention to reduce perceived stress during the COVID-19 pandemic, inducing a positive affective state and lowering perceived stress |
| Oulefki et al. (2022)  [93] | Education and training | To implement an online virtual teaching protocol using VR to teach the neuroanatomy of the brain and spinal cord and to teach and practice neurorehabilitation exercises | During the first year of the COVID-19 pandemic in Algeria | 6 participants recruited among medical staff (radiologists,  doctors, students, and collaborators) (gender and age unspecified) | Quantitative descriptive (Case report) | Self-reported questionnaires:   - Demographic information - Custom questions on the usefulness and best features of the VR diagnostic COVID-19 platform | The VR tool provided a novel visualization for medical practitioners to identify and analyze the change in the shape of COVID-19 |
| Paul et al. (2020) [95] | Treatment  (mental health) | To examine the feasibility, acceptability, and tolerability of a VR-based intervention for an adult with mild depressive disorder (MDD) during the COVID-19 pandemic | In January 2021 in the US | 1 male in his early 40s with a history of depression, who had been treated with psychotherapy  and medication management | Quantitative descriptive (Case report) | Self-reported questionnaires:   - Demographic information - Patient Health Questionnaire-9 (PHQ-9) - Five-question telepresence scale outlined by Nowak and Biocca - Simulator Sickness Questionnaire (SSQ) - Adapted version of the technology acceptance model (TAM) - Custom quantitative questions on acceptability, tolerability, and depression symptoms   Semi-structured interview:   - The Mini International Neuropsychiatric Interview (MINI)   Users’ sessions data:   - Acceptability was assessed using a compliance, and headset use were obtained from the headset | The participant reported that the intervention was feasible, acceptable, and tolerable. His depressive symptoms decreased over a month |
| Petrica et al. (2021)  [102] | Education and training | To explore the adequacy of 360° videos for emergency medicine (EM) education during the COVID-19 pandemic | During the first semester of the academic year 2020–2021 in Romania | 79 medical students (67% female, N=53; age unspecified) | Mixed methods | Self-reported questionnaires:   - Demographic information - Questionnaires, complemented with basic YouTube analytics - Multiple-choice questionnaires (MCQ) and oral examination, contrasting the results with those in 2019–2020 | Students' interest in and acceptance of the 360° videos were high. The 360° videos were effective in teaching EM |
| Rastlon et al. (2021) [99] | Education and training | To test the use of VR -based simulations in simulating pediatric intensive care unit (CICU) clinical scenarios | During the first year of the COVID-19 pandemic in the US | 6 pediatric cardiac critical care physicians  (age and gender unspecified) | Mixed methods | Self-reported questionnaires:   - Demographic information - Custom quantitative items on realism, engagement, usefulness, enjoyment - Open-end questions on the experience | All clinicians successfully navigated the VR environment and met the critical endpoints of the two clinical scenarios |
| Riva et al. (2021) [74] | Treatment  (mental health) | To investigate the effectiveness of a novel self-administered at-home daily VR-based intervention (COVID Feel Good) for reducing the psychological burden experienced during the COVID-19 lockdown in Italy | From June to July 2020 in Italy | 40 participants who had experienced at least two months of strict social distancing measures (62.5% female, N=25; mean age= 30.3, SD =11.7) | Quantitative  non-randomized  Two conditions:   - 360° video using the VR headset (Group Immersive modality) - 360° video using the YouTube app (Group Non-immersive modality) | Self-reported questionnaires :   - Demographic information - Depression Anxiety Stress Scale (DASS-21) - Perceived Stress Scale (PSS) - Beck Hopelessness Scale (BHS) - Social Connectedness Scale (SCS) - Fear of Coronavirus (FCOR) - State–Trait Anxiety Inventory (STAI) - Smith Relaxation State Inventory 3 (SRSI3) - Subjective Units of Distress Scale (SUDS) | Participants exhibited improvements from baseline to post-intervention for depression, stress, general distress, social connectedness, and perceived stress but not for the perceived hopelessness and in perceived fear of coronavirus. These improvements were maintained from post-intervention to the 2-week follow-up |
| Sadeghi et al. (2021) [82] | Education and training | To study the feasibility, effectiveness, and user experience of a VR-based multidisciplinary  heart team meeting to overcome social distancing challenges due  to COVID-19 | During the first year of the COVID-19 pandemic in the Netherlands | 10 cardiothoracic  surgeons and cardiologists (9 male, 1 female; age unspecified) | Quantitative descriptive (Case report) | Self-reported questionnaires:   - Demographic information - Custom quantitative items on feasibility, subjective VR experience and benefits of immersive meetings (ease-of-use, engagement, usefulness, effectiveness, attitude toward use) | There was a positive attitude towards the use of VR as an alternative method for remote conferencing. The participants felt actively involved in the meetings and did not experience visual or auditory distractions from their surroundings. Communication was mentioned to be intuitive and as good as a physical meeting |
| Sampaio et al. (2021) [98] | Treatment  (mental health) | To compare therapists’ use, training with, and perceived benefits of online therapy and the use of VR before versus during COVID-19 | From April to May 2020 in Spain | 50 mental health professionals (78% female, N=39; mean age=39.4) | Quantitative descriptive (Survey) | Self-reported questionnaires:   - Demographic information - Custom quantitative questions on using telehealth and VR to deliver clinical services before and after the COVID-19 pandemic | During the COVID-19 pandemic, mental health therapists indicated that they had used VR technology to help treat their patients |
| Sampaio et al. (2021) [97] | Treatment  (mental health) | To measure how many therapists are using online therapy before vs. during COVID-19, how much training they have received, and their knowledge about legal restrictions on using telepsychology | From April to May 2020 in the US | 768 mental health professionals (92.2% female, N=706; mean age=43.7, SD = 10.9) | Quantitative descriptive (Survey) | Self-reported questionnaires:   - Demographic information - Custom questions on four key domains of using telehealth to deliver clinical services by professionals psychologists before the COVID-19 pandemic: providers’ use of telecommunication modalities; client population characteristics; professional, ethical, and legal/regulatory issues; and telehealth training and practice | Results show that before COVID-19, most therapists only saw their patients in person (e.g., at the therapist’s office), but during the COVID-19 pandemic, nearly all therapists used a wide range of telecommunication technologies to communicate with their quarantined patients, including texting, telephones, video conferences, and even VR |
| Siani et al. (2021) [86] | Treatment  (mental health, physical activity) | To evaluate the impact of VR activities on users under lockdown due to the COVID-19 pandemic and to compare the perceived effectiveness of VR fitness activities with that of non-VR exergames (e.g., Nintendo Wii) | During the first year of the COVID-19 pandemic in the UK | 646 participants  recruited online via social media (the majority were male; mean age range= 30-34) | Quantitative descriptive (Survey) | Self-reported questionnaires:   - Demographic information - Custom quantitative questions on the recreational use of VR during the lockdown period; its impact on their physical and mental health; the Intensity of fitness activities; body weight | VR use has significantly increased during the lockdown period for most participants, who expressed positive opinions on the impact of VR activities on their mental and physical well-being. Self-reported intensity of physical activity was considerably more strenuous in VR users than in console users |
| Silva et al. (2021) [79] | Treatment  (physical activity) | To evaluate the feasibility and potential benefits of  using VR games for home-based rehabilitation of patients with cerebral palsy (CP) during the quarantine period for COVID-19 | Between March and June 2020 in Brasil | 22 individuals with cerebral palsy (CP)  (41% female, N=9; mean age= 17.8, SD= 9.9), and 22 typically developing  individuals (41% female, N=9; mean age= 16.8, SD= 8.4) | Quantitative  non-randomized | Self-reported questionnaires:   - Demographic information - The Rating of Perceived Exertion - Custom quantitative questions on Motivation and satisfaction   Performance task:   - Accuracy and precision of movement, as well as the number of hits and mistakes during the game | All participants were able to engage with the VR therapy remotely, reported enjoying sessions, and improved performance in some practice moments |
| Speidel et al. (2021) [87] | Education and training | To examine how ad hoc digitization has changed the design of digital teaching, the attitudes toward and the capabilities of digital teaching and learning, and the future importance of individual digital teaching elements | Before the COVID-19 pandemic (Time 1) and during the COVID-19 pandemic (Time 2) in Germany | 568 medical students and lecturers (mean age= 35.5, SD=7; gender unspecified)  2019: 163 medicine students  2020: 285 medicine students | Quantitative descriptive (Survey) | Self-reported questionnaires:   - Demographic information - Custom quantitative items regarding the effects of ad-hoc digitization (i.e., digital teaching design; attitude toward and the capabilities of digital teaching; future of digital technologies) | Video-based teaching elements such as videoconferencing and lecture recordings were increasingly used after the COVID- 19 outbreak and considered more significant for future teaching. In  contrast, VR, augmented reality, and 360°-videos are descriptively becoming less important |
| Vlake et al. (2021) [96] | Treatment  (mental health) | To investigate the efficacy of a VR intervention in reducing posttraumatic stress disorder (PTSD), anxiety, and depression in intensive care unit (ICU) COVID-19 survivors | During the first year of the COVID-19 pandemic in the Netherlands | A 57-year-old male treated in ICU due to COVID-19 | Quantitative descriptive (Case report) | Self-reported questionnaires:   - Demographic information - Impact of Event Scale-Revised - Hospital Anxiety and Depression Scale | One week after receiving the VR intervention, levels of PTSD, anxiety and depression had normalized, and stayed normalized until six months after discharge |
| Vlake et al. (2022) [71] | Treatment  (mental health) | To explore the effects of COVID-19 intensive care unit-specific virtual reality (ICU-VR) intervention on mental health and quality of life among COVID-19 survivors | From June 2020 to February 2021 | 89 patients recruited among COVID-19 ICU patients  (37% female, N=16; mean age=58, SD=11) | Quantitative randomized controlled trial  Two conditions:   - Received the ICU-VR intervention (Experimental Group) - Did not receive ICU-VR (Control Group) | Self-reported questionnaires:   - Demographic information - Impact of Event Scale-Revised (IES-R) - Hospital Anxiety and Depression Scale (HADS) - Short-Form 36 (SF-36) and the European Quality of Life, 5 Dimensions (EQ-5D) - Patient Satisfaction Questionnaire and Family Satisfaction with ICU Care tools | ICU-VR was a feasible and acceptable method to improve satisfaction with and rating of ICU aftercare. A low prevalence of psychological distress was observed after ICU treatment for COVID-19 |
| Volodymyrovych  et al. (2021) [89] |  | To evaluate the use of VR and online teaching system among medical students in Ukraine during COVID-19 pandemic. | During the first year of the COVID-19 pandemic in Ukraine | 226 medical students  (82.5% female, N=185; mean age range 22-25 years old) | Quantitative descriptive (Survey) | Self-reported questionnaires:   - Demographic information - Custom questions with 5 options to comprehensively evaluate VR and online teaching system | Mostly students agreed that VR and online teaching compensated the suspension of face-to-face medical education during the COVID-19 and reported that these technologies are best alternatives to physical learning |
| Wagner et al. (2021) [88] | Education and training | To survey pediatric healthcare providers (HCPs) on their preparation for simulation-based medical education of COVID-19 cases | Between April and May 2020 in nineteen English-speaking countries | 234 individual members of the  three largest international pediatric simulation societies from 19 countries (age and gender unspecified) | Quantitative descriptive (Survey) | Self-reported questionnaires:   - Demographic information - Custom quantitative questions on the COVID-19 specific simulation training, - modalities for COVID-19 simulation-based   education, non-COVID-19 simulation-based education, and use of telesimulation | The swift incorporation of disease-specific sessions and the transition of standard education to virtual or hybrid simulation training modes occurred frequently. Frequent modifications to existing simulation programs included the use of VR training |
| Xing et al. (2021) [76] | Education and training | To analyze how to use VR technology to improve  public education on the COVID-19 pandemic | During the first year of the COVID-19 pandemic in China | 80 university students (gender and age unspecified) | Quantitative  non-randomized  Four conditions:   - Group A (Immersive VR) - Group B (Desktop VR) - Group C (Traditional education group) - Group D (Control group) | Self-reported questionnaires:   - Demographic information - Single choice, multiple choice and judgment questions on COVID-19 knowledge | VR was an effective educational tool for the COVID-19 crisis fundamental knowledge |
| Yahara et al. (2021) [91] | Treatment  (mental health) | To examine the effectiveness and safety of reminiscence using immersive VR in patients with mild cognitive impairment (MCI) | From February to April 2020 in Japan | 2 patients with MCI (a 80-yr-old male and a 92-yr-old female) | Quantitative descriptive (Case report) | Self-reported questionnaires:   - Demographic information - State-Trait Anxiety Inventory (STAI) - Numerical Rating Scale (NRS) on the degree of side effects (nausea, dizziness, and headache) and satisfaction with the VR session - Zarit Caregiver Burden Interview - (J-ZBI_8) - Dementia Behavior Disturbance Scale (DBD13) - Apathy scale - Tokyo University Style Observation Rating Scale (TORS) - Pittsburgh Rehabilitation Participation Scale (PRPS) | Immersive VR reminiscence could reduce anxiety and the burden of care without serious side effects. Its effect might be almost as effective as those of face-to-face one |
| Yang et al. (2021) [92] | Treatment  (mental health) | To examine how 360° virtual tours can help to reduce people’s psychological stress during the COVID-19 pandemic | During the first year of the COVID-19 pandemic in China | 235 participants who were collected face-to-face at shopping  malls (majority female; mean age range=  18-25) | Quantitative descriptive (Survey) | Self-reported questionnaires:   - Demographic information - Sense of presence - Enjoyment - Telepresence - Involvement - 360° virtual tour experience - Symptoms of stress | Among the factors, enjoyment shows the highest effect on satisfaction with the 360° virtual tour experience and stress reduction |
| Yang et al. (2022)  [94] | Treatment  (physical activity, mental health) | This research aimed to find out the role of virtual reality fitness (VRF) for  behavior management during the COVID-19 pandemic | Between January and March 2020 in China | 4300 participants who were Chinese residents who  were in home isolation during the early pandemic in China (51% female, N=2195; mean age range  20–29 years old) | Quantitative descriptive (Survey) | Self-reported questionnaires:   - Demographic information - Physical activity-related questions (e.g., frequency of physical activity during the COVID-19 lockdown) - VRF for behavior and attitude management during the COVID-19 questions (e.g., if VRF is helpful to maintain psychological well-being) | VRF play a substantial role in  improving the overall quality of life and positively influencing behavior and attitude during the COVID-19 crisis |
| Zhang et al. (2021) [75] | Education and training | To develop a VR-based training program and further verify the effect of the program on improving the response capacity of emergency reserve nurses | From May to July 2020 in China | 120 nurses (91.7% female, N=55; most aged under 30) | Quantitative  non-randomized  Two conditions:   - VR training in combination with skills training   (Experimental Group)   - Conventional training of emergency response - (Control Group) | Self-reported questionnaires:   - Demographic information - Disaster Preparedness Evaluation Tool   Knowledge assessment:   - Knowledge and technical skills regarding responses to fulminate respiratory infectious diseases, as well as the capacity of emergency care - Assessment of theories and skills | The VR simulation training in combination with technical skills training can improve the response capacity of emergency reserve nurses as compared with the conventional training |
| Zhang et al. (2020) [85] | Treatment  (mental health) | To explore the role of VR exposure therapy (VRET) in the intervention of psychiatric illnesses with chief complaints of fear of COVID-19 | In June 2020 in China | 3 patients with chief complaints of fear of COVID-19 (two 41-yr-old males and a 55-yr-old female) | Quantitative descriptive (Case report) | Self-reported questionnaires:   - Demographic information - Hamilton Anxiety Rating Scale (HAMA) - Fear of COVID-19 Scale (FCV-19S) | VRET intervention significantly reduced the related symptoms caused by fear of COVID-19 |
