# Supplementary material for: Virtual Reality Applications in Medicine During the COVID-19 Pandemic: Systematic Review
Source: JMIR Serious Games. 2022 Oct 25;10(4):e35000. doi: 10.2196/35000 (PMC9605086; doi:10.2196/35000)
Supplement: Multimedia Appendix 3 [file games_v10i4e35000_app3.docx]

**Multimedia Appendix 3. Virtual reality characteristics.**

| **Study** | **Immersion** | **Type of HMD** | **Content** | **Site of use** | **User mode** | **Time** | **Description of the experience** |
| --- | --- | --- | --- | --- | --- | --- | --- |
| Beverly et al. (2022) [80] | High  (Immersive VR) | Standalone  (Oculus Go or Pico G2 4k) | 360° videos | Face-to-face | Single-user | 1 session,  3 minutes | *Tranquil-Cine VR Simulation,* a 360° scene of a local nature preserve |
| Birrebach et al. (2021) [68] | High  (Immersive VR) | PC-based  (Oculus Rift S) | Virtual environments | Face-to-face | Single-user | 1 session,  time unspecified | *COVID-19 VR Strikes Back (CVRSB)*, a VR experience showing the taking of a nasopharyngeal swab |
| Buyego et al. (2021) [100] | High  (Immersive VR) | PC-based  (HTC Vive) | A hybrid of VR environments and 360° videos | Face-to-face | Single-user | 2 sessions,  45 minutes each | A VR experience on wearing and stripping Personal Protective Equipment (PPE) |
| Campo-Preito et al. (2021) [90] | High  (Immersive VR) | PC-based  (HTC Vive Pro) | VR video games  (COTS) | Face-to-face | Single-user | 2 sessions,  10-12 minutes each | Two VR games downloaded from the Steam Store (i.e., *Box VR* and *NVIDIA VR Fun House*) |
| Cecil et al.  (2021) [73] | High  (Immersive VR) | PC-based  (HTC Vive) | Virtual environments | Face-to-face | Single-user | 1 session,  time unspecified | Three training environments developed for the  first responders training focused on the COVID-19 pandemic |
| De Ponti et al. (2020) [48] | Low  (Desktop VR) |  | Embodied  virtual agents | Remotely | Single-user | 6 sessions,  20 minutes each | Twenty-one patient-based clinical scenarios on the *Body Interact*™ *Clinical Education* platform |
| Flo et al.  (2021) [104] | Low  (Desktop VR) |  | Embodied  virtual agents | Remotely | Single-user | 2 sessions,  time unspecified | A virtual COVID-19 patient and a virtual surgical trauma patient on the *Body Interact*™ virtual simulator |
| Garcia et al.  (2021) [70] | High  (Immersive VR) | Standalone  (Pico G2 4K) | Virtual environments, VR video games (ad hoc), 360° videos | Face-to-face | Single-user | 56 sessions,  6 minutes each | *EaseVRx* combines biopsychosocial education,  diaphragmatic breathing training, relaxation response exercises, and executive functioning games to provide a mind–body approach toward living better with chronic pain |
| Guichet et al. (2021) [83] | Low  (Desktop VR) |  | Virtual environments | Remotely | Multi-users | 1 session,  time unspecified | A VR Meeting Hall dedicated to the pre-interview social designed in *Mozilla Hubs* using Mozilla’s open-source Hubs environment |
| Herbst et al.  (2021) [101] | Low  (Desktop VR) |  | Embodied  virtual agents | Remotely | Single-user | 1 session,  30 minutes | A patient-based clinical scenario on counseling caregiver avatars regarding typical behavior concerns for a 3-year-old child |
| Jeong et al.  (2022)[77] | High  (Immersive VR) | Standalone  (Oculus Quest 2) | Virtual environments | Face-to-face | Single-user | 1 session.  15 minutes | A COVID-19 related virtual scenario presenting clinical cases of patients with respiratory infections |
| Kang et al.  (2020) [78] | Low  (Desktop VR) |  | Embodied  virtual agents | Face-to-face | Single-user | 1 session,  15 minutes | A patient-based clinical scenario of a child with mild intermittent asthma was accessed through the *vSim* platform. The patient in the presented scenario was a 5-year-old girl who experienced asthma symptoms and was admitted to the emergency room |
| Kolbe et al.  (2021) [49] | High  (Immersive VR) | Standalone  (SootheVR) | Virtual environments  and VR video games (ad hoc) | Face-to-face | Single-user | 1 session,  10 minutes | *AppliedVR* software experiences. Three categories of experiences: guided meditation; exploration of natural environments; cognitive stimulation games |
| Leung et al.  (2021) [84] | High  (Immersive VR) | Mobile  (e.g., Google Cardboard) | 360° videos | Face-to-face | Single-user | Unspecified | 360° videos on ureteric reimplantation, penile fracture, and open cystostomy |
| Liu & Butzlaff (2021) [72] | Both | Cardboard | 360° photos | Face-to-face versus remotely | Single-user | 1 session,  90 minutes | Students created a tour from a Ricoh Theta 360° photos |
| Mottelson et al. (2021) [69] | High  (Immersive VR) | Standalone  (Oculus Quest 1 and 2) | Virtual environments | Remotely | Single-user | 1 session,  time unspecified | Three virtual scenes about COVID-19 vaccination |
| Nijland et al. (2021) [81] | High  (Immersive VR) | Standalone  (Oculus Go) | 360° videos | Face-to-face | Single-user | Minimum of 1 session,  10 minutes each | *VRelax*, a high-quality immersive 360-degree video of calming natural environments. Options include walking on a  beach and underwater swimming with wild dolphins. |
| Oulefki et al. (2022) [93] | High  (Immersive VR) | PC-based  (Oculus Rift S) | Virtual environments and 3D images | Face-to-face | Single-user | 1 session,  7 minutes | VR scenarios which depict the volume-rendered COVID-19 lesions inside 3D lungs in a virtual room |
| Paul et al.  (2020) [95] | High  (Immersive VR) | Standalone  (Limbix VR) | 360° videos | Remotely | Single-user | 4 sessions,  10 minutes each | 360° videos chosen from 360° videos already accessible on YouTube. These 360° videos were uploaded onto the headset, and each video was sorted into different categories, including animals; sports, dance, or arts |
| Petrica et al. (2021) [102] | Low  (Desktop VR) |  | 360° video | Remotely | Single-user | 1 session,  time unspecified | 360° videos located in a hospital emergency department. The videos covered the assessment, management, and treatment of patients with diverse specific pathologies |
| Rastlon et al. (2021) [99] | High  (Immersive VR) | Standalone  (Oculus Quest) | Embodied  virtual agent | Face-to-face | Single-user | 1 session,  4-8 minutes | Two patient-based clinical scenarios created using the medical VR simulation *SimX platform* |
| Riva et al.  (2021) [74] | Low  (Desktop VR)  vs. High  (Immersive VR) | Mobile  (like Google Cardboard) | 360° video | Remotely | Single-user | 7 sessions,  10 minutes each | *The Secret Garden,* a 10-min computer graphic 360° video for relaxation training. Well-being psychologists have written this experience to mimic the structure and the experience of walking in a Japanese garden, providing the visual and auditory natural elements available outdoors |
| Sadeghi et al. (2021) [82] | High  (Immersive VR) | PC-based  (Varjo VR-1 and Oculus Rift S) | Virtual environments | Face-to-face | Multi-users | Unspecified | VR meetings on the *MeetinVR* platform. During the VR meetings, a coordinator provided heart team participants with anonymized medical images of a patient who already had been discussed in an earlier heart team meeting |
| Silva et al.  (2021) [79] | Low  (Desktop VR) |  | VR video games  (ad hoc) | Remotely | Single-user | 2 sessions,  3-9 minutes each | A serious game created using the platform *MoveHero*. Users are presented with several spheres falling down the computer screen, with a musical rhythm to increase engagement |
| Vlake et al.  (2021) [96] | High  (Immersive VR) | Unspecified | 360° videos | Face-to-face | Single-user | 2 sessions,  time unspecified | COVID-19 intensive care unit-specific virtual reality (ICU-VR) intervention. Six scenes on COVID-19 ICU treatments (e.g., mechanical ventilation in a prone position) |
| Vlake et al.  (2022) [71] | High  (Immersive VR) | Standalone (Oculus Go) | 360° videos | Face-to-face | Single-user | 1 session, 14 minutes | As above |
| Xing et al.  (2021) [76] | Low  (Desktop VR)  vs. High  (Immersive VR) | PC-based  (HTC Vive) | Virtual environments | Face-to-face | Single-user | 1 session,  20 minutes | Two virtual environments: a medical center and a clinical case scenario |
| Yahara et al.  (2021) [91] | High  (Immersive VR) | Standalone  (Oculus Go) | 360° photos | Remotely | Single-user | 1 session,  10 minutes | *Wander,* a 360° photos travel app that can view Google map street view to search for the place they want to visit |
| Yang et al.  (2021) [92] | Low  (Desktop VR) |  | 360° video | Face-to-face | Single-user | 1 session,  3 minutes | A 360° video available on YouTube (i.e., *VR Gondola Ride in Venice!*) |
| Zhang et al.  (2021) [75] | Low  (Desktop VR) |  | Virtual environments | Unspecified | Single-user | 12 sessions,  time unspecified | Four virtual scenes, including prehospital management, fever clinic reception, intensive care unit (ICU), and isolation ward |
| Zhang et al.  (2020) [85] | High  (Immersive VR) | PC-based  (HTC VIVE Pro EyeProfessional) | Unspecified | Face-to-face | Single-user | Unspecified | COVID-19 related VR scenarios on different situations, such as touching stained door handle which may have viruses, watching pandemic news, watching frontline health care workers |
